# High-resolution genetic mapping of maize pan-genome sequence anchors

**DOI:** 10.1038/ncomms7914

**Published:** 2015-04-16

**Authors:** Fei Lu, Maria C. Romay, Jeffrey C. Glaubitz, Peter J. Bradbury, Robert J. Elshire, Tianyu Wang, Yu Li, Yongxiang Li, Kassa Semagn, Xuecai Zhang, Alvaro G. Hernandez, Mark A. Mikel, Ilya Soifer, Omer Barad, Edward S. Buckler

**Affiliations:** 1 Institute for Genomic Diversity, Cornell University, Ithaca, New York 14850, USA; 2 United States Department of Agriculture/Agricultural Research Service, Ithaca, New York 14850, USA; 3 Institute of Crop Science, Chinese Academy of Agricultural Sciences, Beijing 100081, China; 4 International Maize and Wheat Improvement Center (CIMMYT), Nairobi 00621, Kenya; 5 International Maize and Wheat Improvement Center (CIMMYT), Apdo 6-641, 06600 Mexico DF, Mexico; 6 Roy J. Carver Biotechnology Center, University of Illinois at Urbana-Champaign, Urbana, Illinois 61801, USA; 7 Department of Crop Sciences, University of Illinois at Urbana-Champaign, Urbana, Illinois, 61801, USA; 8 NRGENE, 3 Golda Meir St. Park HaMada, Ness Ziona 7403648, Israel

## Abstract

In addition to single-nucleotide polymorphisms, structural variation is abundant in many plant genomes. The structural variation across a species can be represented by a ‘pan-genome’, which is essential to fully understand the genetic control of phenotypes. However, the pan-genome’s complexity hinders its accurate assembly via sequence alignment. Here we demonstrate an approach to facilitate pan-genome construction in maize. By performing 18 trillion association tests we map 26 million tags generated by reduced representation sequencing of 14,129 maize inbred lines. Using machine-learning models we select 4.4 million accurately mapped tags as sequence anchors, 1.1 million of which are presence/absence variations. Structural variations exhibit enriched association with phenotypic traits, indicating that it is a significant source of adaptive variation in maize. The ability to efficiently map ultrahigh-density pan-genome sequence anchors enables fine characterization of structural variation and will advance both genetic research and breeding in many crops.

## Abstract (web summary)

Structural variations in crop genomes are thought to be responsible for significant differences in phenotype and they can be well-represented by a ‘pan-genome’. Here, Lu *et al.* develop an approach to genetically map pan-genome sequence anchors using 14,129 inbred lines of maize, showing structural variation is a significant source of adaptive variation.

## Graphical abstract

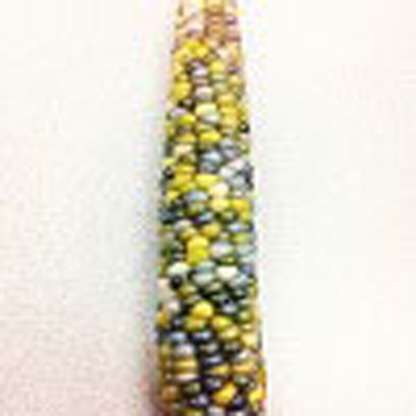

Genome duplication^1^ and transposable elements^2^ (TEs) are important driving forces behind plant genome evolution, and have generated the complex genomes found in many major crop species^3^,^4^,^5^,^6^,^7^. These complex genomes contain tremendous structural variation (SV), in the form of copy number variation (CNV), presence/absence variation (PAV, an extreme form of CNV), inversion and translocation. In humans, CNV has a limited influence on disease susceptibility and explains only a minority of the ‘missing heritability’^8^,^9^. However, in major crop species, CNV is much more prevalent^10^,^11^, and thus is much more likely to significantly have an impact on phenotypic variation. For example, plant disease defense genes often display CNV and frequently colocalize with other CNVs^12^,^13^. Furthermore, read depth variation is over-represented in genome-wide association study (GWAS) hits for multiple traits in maize^14^. These observations suggest that CNV plays an important role in phenotypic variation.

To characterize CNVs, an ideal system is the pan-genome, a representation of both the core genome (collinear genome) and the variably distributed genome (SVs) of a species^15^. The pan-genome can be constructed by comparing multiple genomes derived from *de novo* sequence assembly (Fig. 1). As a result of the falling cost of sequencing, it is now possible to sequence crop varieties on an unprecedented scale in both depth and sample size. However, because of the repetitive nature of complex genomes, prevalent alignment ambiguity hinders accurate read contiging and confounds pan-genome assembly. In addition, a large proportion of genomic fragments absent from the reference cannot be placed on the pan-genome via alignment. Therefore, for species with complex genomes, sequence alignment alone is insufficient to build high-quality pan-genomes. However, the availability of a set of ultrahigh-density genetic anchors would be extremely helpful to the pan-genome construction. These genetic anchors could be used either to evaluate assembly quality or, even better, to direct *de novo* assembly of individual genomes^6^. Genotyping-by-sequencing (GBS)^16^, a reduced representation approach, can efficiently generate abundant single-nucleotide polymorphisms (SNPs) for a large number of individuals of a species. It is also a cost-effective source of sequence tags that can be used as genetic anchors to direct contig/scaffold assembly and to map genomic fragments absent in the reference. In this study, we developed an efficient and accurate approach to genetically map ultrahigh-density sequence anchors, which will be a valuable tool for ongoing pan-genome construction. This approach is most powerful with the large sample size of individuals afforded by GBS. This analysis was conducted in maize, the largest production crop in the world, which is also a model species for complex genomes. Mapping sequence anchors in maize provides an effective example for other species.

Maize is among the major crop species that exhibit the highest amounts of SVs. Between any two maize varieties, about half of the genome is not shared because of the high level of TE activity during maize evolution^17^,^18^. The B73 maize reference genome confirmed that nearly 85% of the B73 genome consists of TEs^6^, which went through repeated cycles of expansion and loss^19^. These elements increased the maize genome by 50% in the last 3 million years^20^. Moreover, the 35% genome size difference between maize and its close relative *Zea luxurians* is accounted for by TEs^21^. In addition to the contribution of TE sequences themselves to SV, numerous gene sequence fragments have been relocated or duplicated by Pack-MULEs^22^, helitrons^23^, retrotransposition^24^ or duplication^25^. A recent study reported 8,681 novel maize transcripts absent in B73 (ref. 26), a number equivalent to 14% of the transcripts in the reference genome. Relative to the majority of the genome that is kept in flux by these processes, the stable, core genome appears to be a rather small proportion of the pan-genome. Hence, a single reference genome is woefully insufficient to represent all genomic contents for maize.

To facilitate building a maize pan-genome that includes *de novo* assemblies of diverse maize varieties, we developed an effective approach to produce high-resolution pan-genome sequence anchors. By genetically mapping 26 M GBS tags in 14,129 maize inbred lines, ∼4.4 M tags were identified as genetic anchors of maize pan-genome, 1.1 M of which were PAVs. These PAVs exhibited enriched associations with multiple traits, suggesting that they play an important role in controlling phenotypic variation. These high-quality pan-genome anchors will be very helpful to direct *de novo* genome assembly and characterize SVs in maize.

## Results

### Initial genetic mapping of GBS tags

**Figure 2:**
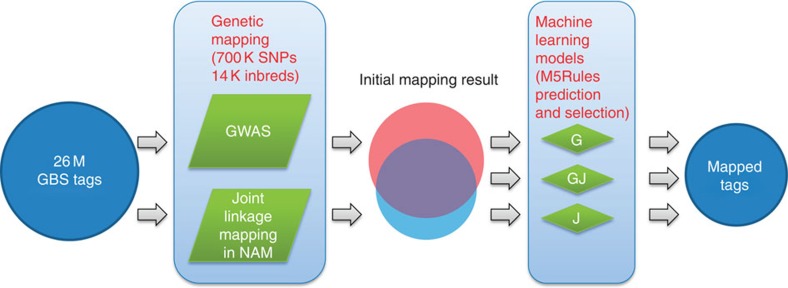
Anchoring GBS tags using genetic mapping approaches combined with ML algorithms. Two genetic mapping approaches, GWAS and joint linkage mapping in NAM, are performed to map GBS tags. Since some tags are mapped by the two methods and others are mapped by both, three corresponding ML models are trained to predict and select accurately mapped tags.

Using GBS, we sequenced a large collection of 14,129 maize inbred lines. These inbred lines are the most comprehensive and representative set of temperate, subtropical and tropical germplasm used in maize genetic research to date. About 1.3 trillion bp of sequence was generated with an average depth of 0.3 reads per site per sample. Using the GBS bioinformatic pipeline^27^,^28^ (Supplementary Fig. 1), a total of 26,436,248 GBS tags were identified. From these tags, 681,257 SNPs were discovered and scored across all of the maize lines^29^. In order to construct a framework for the maize pan-genome, genetic mapping approaches combined with machine-learning (ML) algorithms were used to anchor GBS tags from the 14 K inbreds to B73 reference genome coordinates (Fig. 2).

The GBS tags were genetically mapped by testing for associations between the presence/absence pattern of each tag and individual GBS SNP genotypes across the 14-K maize inbreds, where the position of the most significant SNP was taken to be the the genetic position of the tag (Supplementary Fig. 2). Two genetic mapping approaches, GWAS and joint linkage mapping in nested association mapping population (NAM)^30^, were performed to map tags with a total of 18 trillion tests. There were 14,975,910 and 6,890,040 tags mapped by GWAS (*P* value <1E−6) and joint linkage mapping (*P* value <0.05), respectively. Uniquely aligned B73 tags (UABTs) were used to assess mapping accuracy, with perfect accuracy consisting of identical genetic and physical positions. In the initial mapping results, only 63.9% and 68.9% of tags were mapped to correct chromosome for the two methods, respectively (Supplementary Fig. 3). This low initial accuracy was due to (1) the lack of correction for population structure (for computational speed), (2) the loose *P* value threshold for joint linkage mapping and (3) the repetitive nature of some of the tags. However, several valuable attributes were collected to model mapping accuracy.

### Generating pan-genome anchors using ML models

To obtain high-quality pan-genome anchors, we developed ML models to predict the accuracy and identify accurately mapped tags as sequence anchors. A total of 16 attributes were collected to predict distances between tags’ physical positions (alignment positions) and their genetic mapping positions (Supplementary Table 1). ML models were trained on UABTs mapped by GWAS, by joint linkage mapping, and by both. Since mapping tags by GWAS does not require designed populations derived from controlled crosses (for example, NAM) and is more likely to be widely used in other species, we use GWAS mapping results to illustrate the ML prediction and filtering.

**Figure 3:**
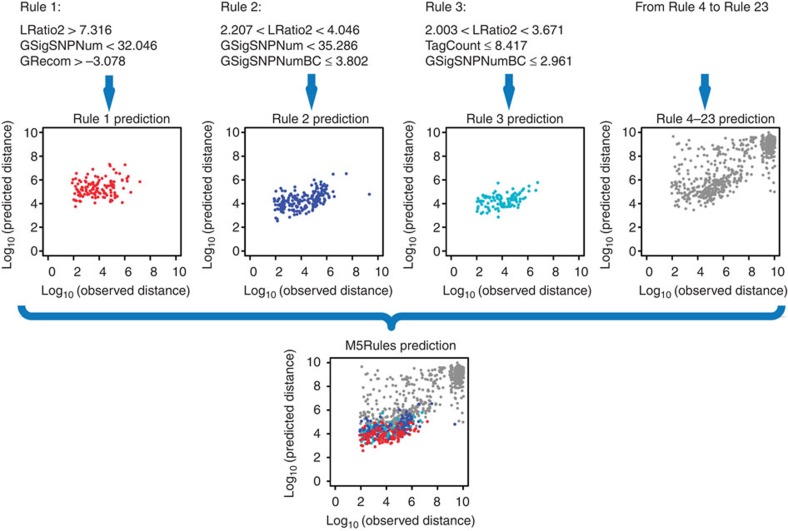
Predicting the distance between GWAS mapping position and observed position of UABTs by M5Rules_G. A total of 1,000 predictions are plotted. M5Rules generates 23 rules on the basis of the attributes. The data set is divided into subsets by the rules. Linear regression is then performed in each subset. Predictions of these subsets are merged into a final data set.

Multiple ML algorithms, including decision tree, association rule and support vector machines, were tested on UABTs mapped by GWAS using nine attributes (Supplementary Table 2). Mapping accuracy (distance between the physical and genetic positions of each UABT) was used as the dependent variable. On the basis of the Pearson’s correlation coefficient (*r*) and mean error between observation and prediction, M5Rules^31^,^32^ performed best (Supplementary Fig. 4). The M5Rules model trained for tags mapped by GWAS was designated as ‘M5Rules_G’. The nine attributes showed various levels of importance for prediction (Supplementary Fig. 5). A total of 23 rules and linear models were generated in M5Rules_G (Fig. 3 and Supplementary Table 3), classifying the tags into 23 subsets. In each subset, linear regression was performed. A moderately high value of *r*^2^ (0.68) between observation and prediction was derived from the merged data set, which enabled selection of accurately mapped tags (Supplementary Table 4).

To examine the robustness of this combined approach (genetic mapping plus ML), several additional factors, including sequence depth of inbreds having the tag, and the amount of missing/imputed genotypes around the genetic positions of mapped tags, were also assessed for their potential impact on mapping accuracy. Results showed that these factors had trivial effects (*r*^2^<0.01, Supplementary Fig. 6). As expected, population structure had a dramatic impact on mapping accuracy: less accurately mapped tags were more likely to correlate with the population structure. However, the effect of population structure on mapping accuracy was well captured by multiple ML attributes, which were the key predictors in the M5Rules_G model (Supplementary Fig. 7). In addition, genetic diversity was also evaluated for its effect on mapping performance. Tag GWAS mapping was tested in two populations (each with *n*=400) with different levels of diversity. One consisted of two NAM families, while the other was a random subset of individuals from the Ames association panel^29^. Owing to the increased within-population genetic diversity and faster decay of linkage disequilibrium, genetic mapping in the Ames subset provided higher resolution (Supplementary Fig. 8). After building ML models, many more sequence anchors at the desired level of accuracy can be selected from Ames mapping results (Supplementary Tables 5 and 6). This indicates that diversity is a key factor influencing tag genetic mapping performance.

In addition to the M5Rule_G model, two M5Rules models, M5Rules_J and M5Rules_GJ, were trained for tags mapped only by joint linkage mapping (M5Rules_J) and by both GWAS and joint linkage mapping (M5Rules_GJ), with different sets of attributes for each (Supplementary Table 1). Compared with M5Rule_G, the M5Rules_GJ model with a few more attributes increased *r*^2^ between prediction and observation from 0.68 to 0.72 and therefore improved the resolution of the resultant subset of tags selected as sequence anchors. However, it should be noted that the M5Rule_G model alone, which did not require a specially constructed population, was capable of producing high-resolution anchors (Supplementary Tables 3 and 7).

**Figure 4:**
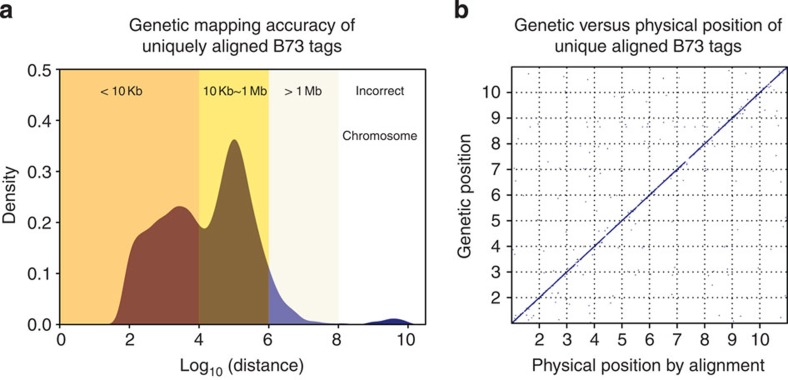
Genetic mapping accuracy of uniquely aligned B73 tags (UABTs) in 4.4-M mapped tags. A total of 20,000 randomly chosen UABTs were used as a quality control to evaluate the performance of genetic mapping. (**a**) The distribution of distance between genetic position and physical position (alignment position) of UABTs. Positions are transformed with an equation of pos=chromosome × 1E9+pos. (**b**) The scatter plot of genetic position against physical position of UABTs. The *x* and *y* axes are maize chromosomes.

The three M5Rules models were applied to initial mapping results. To keep accurately mapped tags, 100, 50 and 100 kb were set as the thresholds for M5Rules_GJ, M5Rules_G and M5Rules_J, respectively (Supplementary Table 8). A total of 4,436,135 high-resolution tags were selected. Of these, 406,019 were UABTs, 99.1% of which were assigned to the correct chromosome, 95.0% within 1 Mb of their actual site and 54.8% within 10 kb (Fig. 4). Of the 4.4 M anchor tags, 946,711 were B73 tags (present in B73 samples): 94% of these B73 tags had a unique perfect alignment match to the reference (Supplementary Fig. 9). This indicates that the 4.4-M mapped tags were enriched for low copy sequences. Therefore, the majority of mapped tags appear to qualify as unique sequence anchors for the maize pan-genome.

### Accuracy of sequence alignment in maize

Sequence alignment underlies genome assembly quality, which is fundamental for pan-genome construction. To evaluate the validity of sequence alignment against the complex maize genome, a GBS library with 95 highly diverse maize inbreds was sequenced using MiSeq 2 × 250 bp paired end sequencing. Alignment positions of reads of various length were compared with their genetic positions obtained from 4.4-M sequence anchors (Supplementary Fig. 10). Taking into account that a sequence anchor has a 98.6% chance (Fig. 4a) to be in the 10-Mb region of its actual position, the alignment with a physical position in the 10-Mb region of read genetic position was arbitrarily considered as a correct alignment. We found that reads with a range from 150 to 300 bp, which is the standard read length of Illumina machines, had about a 20% chance to be incorrectly aligned in maize (Supplementary Fig. 11). This illustrates the challenge of genome assembly and SNP discovery in species with complex genomes.

### Identifying PAV anchors

**Figure 5:**
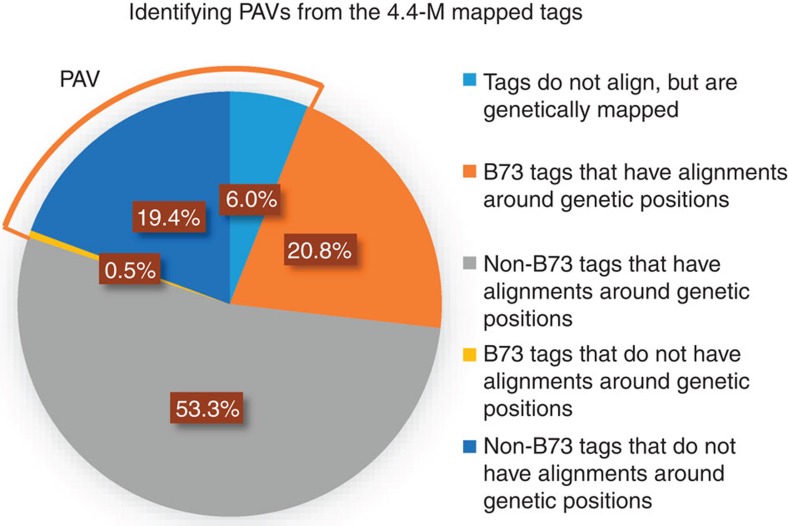
Identification of PAVs from the 4.4-M mapped tags. The 4.4-M mapped tags were aligned to the B73 reference genome and the physical positions were compared with their genetic positions. If a tag did not align to the reference or did not have alignment within the 10-Mb region of its genetic position, then it would be considered as a PAV tag. The B73 tags are those that have at least one perfect match to the reference genome.

We found a considerable number of mapped tags that either did not align to the reference at all, or did not align within 10 Mb of their genetic positions (Fig. 5). These tags were defined as PAVs, which essentially tagged genomic sequence absent in orthologous regions in the B73 reference genome. About 0.5% of B73 tags did not have an agreement between genetic position and physical position. This is probably because of two reasons: (1) the same tag is at another position in non-B73 genomes because of translocation or duplication; (2) there is about a 1.4% chance that genetic positions of the 4.4-M tags are not in a 10-Mb region of their actual positions (Fig. 4). However, we were not able to differentiate the two scenarios. Within the 4.4-M tags, a total of 1,147,512 (26%) tags were classified as PAVs. This suggested that B73 contains ∼74% of the low copy sequence of maize, which reflected an earlier estimate of 70% (ref. 33). We found that PAVs were more often present in pericentromeric regions (Supplementary Fig. 12), which might be due to high amounts of TE and relatively poor assembly around centromeres. In the human genome, there is also higher proportion of PAVs in pericentromeric regions^34^. We also found that PAV density was positively correlated with repeat density, but negatively correlated with recombination rate and gene density. This suggests that repetitive sequences are a major contributor to PAV in maize.

To initiate construction of the maize pan-genome, we recently deeply sequenced and assembled the maize inbred line CML247, a valuable line for maize breeding because of its high disease resistance. The 4.4-M genetic anchors turned out to be a powerful resource to assess the quality of the CML247 assembly (Supplementary Fig. 13). To validate the 1.1-M PAV tags absent in the B73 reference, a total of 200 high-quality scaffolds from the CML247 assembly were compared with their orthologous regions in B73. By aligning 1.1-M PAV tags to the 200 CML247 scaffolds, we found that 89% of PAV anchors tagged genomic sequences present in CML247 but absent in B73 (Supplementary Fig. 14).

### PAV and phenotypes

To investigate the contribution of PAVs to phenotypic variance, we conducted GWAS for four traits (days to silking, days to anthesis, plant height and ear height). The 700-k SNPs were used to perform GWAS in 2,661 maize inbred lines (Supplementary Data 1 and 2). Since the PAVs were genetically mapped via their co-segregation with SNPs, the SNPs where the PAVs were mapped served as proxies to estimate their genetic effect. Accordingly, SNPs were divided into 228,620 PAV SNPs (associated with PAVs) and 452,637 ordinary SNPs (not associated with PAVs). In the GWAS analysis, the population structure was well accounted for and the positive control loci (genes known to affect flowering time in maize) had GWAS hits (Supplementary Figs 15 and 16).

Both PAV SNPs and ordinary SNPs generated significant *P* values. Since PAV tags identified by genetic mapping approaches were biased towards high-frequency ones, the ordinary SNPs had a larger proportion of low-frequency alleles relative to PAV SNPs (Supplementary Fig. 17). To maintain equal statistical power, we controlled for minor allele frequency (MAF) of ordinary SNPs and PAV SNPs before comparing their *P* values. After filtering out SNPs with MAF<0.095, the 66,998 ordinary SNPs and 117,917 PAV SNPs had equal MAF distributions, in which the median MAF was 0.25 for both (Supplementary Fig. 18).

**Figure 6:**
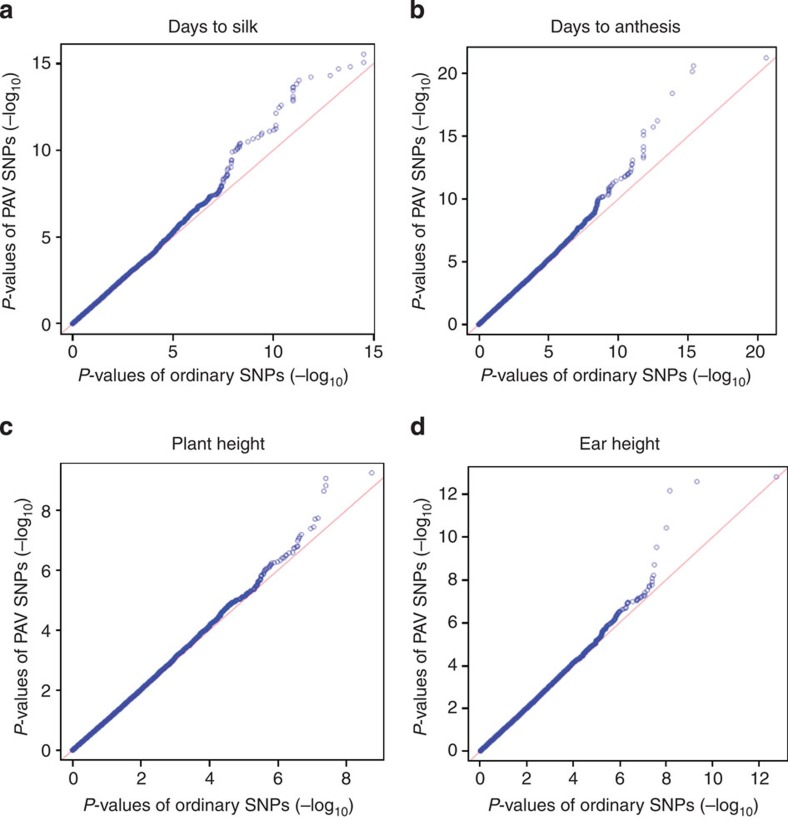
QQ plots showing PAV SNPs are enriched for the significant GWAS hits. For the large amount of insignificant SNPs, the PAV SNPs and ordinary SNPs did not show differentiated distributions; while for those significant GWAS hits, PAV SNPs exhibited enriched associations with traits.

PAV SNPs were enriched for the significant GWAS hits relative to the ordinary ones (Fig. 6 and Supplementary Table 9), suggesting that PAVs have an important role in generating phenotypic variation. Taking into account that 62.9% of PAV SNPs were within genes and 54.8% of PAV tags were in 10-kb flanking regions of these SNPs, these SVs might alter gene expression by modifying gene regulation, as was suggested by an earlier study^35^.

## Discussion

Analysing an unprecedented number of inbred lines in maize, we developed effective genetic mapping approaches combined with ML algorithms to map millions of high-quality sequence anchors for the maize pan-genome. We also found that PAVs play an important role in controlling phenotypic traits. Along with a previous observation that RDVs were over-represented for significant associations with traits^14^, we hypothesize that CNVs are a significant source of adaptive variation in maize. The large numbers of CNVs represent a rich and potentially underutilized resource for maize-breeding programmes.

As we mentioned, a pan-genome is an ideal system to capture CNVs and other structure variations. One efficient way to build the pan-genome is to *de novo* assemble individual genomes of representative varieties/accessions. However, it is still challenging to *de novo* assemble complex genomes^36^. Without a powerful quality-control, many misassembled contigs would be incorrectly interpreted as SVs. Genetics is the true proof of sequence assembly. The genetic mapping approach developed here can produce a high-density genetic grid to validate sequence placement in contigs/scaffolds and help put them to the right place. *De novo* assembly of individual genomes plus millions of genetic anchor points should be a great combination to effectively construct pan-genome for species with complex genomes.

One alternative to build a pan-genome is to skim sequence multiple varieties and assemble reads that do not align to the reference genome. This might work for species with simple genomes. However, it is unlikely to adequately capture SVs in complex genomes, such as maize. This is due to two reasons: (1) although ∼95% of reads from a nonreference inbred can be aligned to the B73 reference genome (default parameters of alignment tools), many of them align to the wrong place. Given that only about half of genome sequences are orthologous when comparing two maize inbreds^17^,^18^, >40% of the aligned reads are from regions of SV and are expected to be misaligned. This suggests that pan-genome sequences equivalent to 40% of maize genome size are missing from a nonreference inbred. The 5% of reads that do not map cannot be well assembled, as they are noncontiguous, sparse and shallow. (2) With respect to sequence depth of skim sequencing, the depth follows a Poisson distribution, which means that reads are not evenly distributed across the genome. Skim sequencing cannot guarantee that reads cover the genome completely. Therefore, many genomic regions will be inadequately covered by skim-sequencing, which will lead to more missing sequence in the pan-genome.

It should be noted that the genetic mapping approach and ML modelling work ideally for species with high-quality reference genomes, since the distance of genetic position and physical position of tags is required as the response variable in the ML model. Here, high quality means that the reference assembly is at a chromosome/pseudomolecule scale. For those species whose reference genomes consist most of scaffolds, the genetic mapping approach can still work. However, training tags should be selected from those relatively larger scaffolds where these tags are aligned to and genetically mapped as well. As long as distance values from a few thousand tags are collected, ML can be performed to select those accurately mapped tags as pan-genome anchors. We also note that this may be an effective way to improve the reference genome, since these anchors provide genetic linkage between different scaffolds/contigs. Researchers will be able to connect the scaffolds/contigs using these genetic links, instead of developing many mapping populations to producing genetic markers.

The 4.4-M sequence anchors developed in this study will aid the construction of an accurate maize pan-genome that can then be used to characterize CNVs. In response to increasing demand for food security and biofuels, more species will be sequenced in the near future and added to the 95 available plant genomes (CoGepedia, https://genomevolution.org/wiki/index.php/Sequenced_plant_genomes). It is anticipated that characterizing SNPs and SVs via pan-genome projects and evaluating their genetic effects will be very important for plant genetic research over the coming decade^37^. Since many plants have complex genomes, these approaches we developed here will be quite valuable to improve genome assembly and explore genomic diversity in many other crops.

## Methods

### Sample collection and genotyping

A broad collection of maize inbred lines were sampled in this study (Supplementary Data 3), including the NAM population^38^, maize inbred lines conserved at the USDA Plant Introduction extension in Ames (IA) or the Ames association panel^29^, maize inbred lines from the International Maize and Wheat improvement Center (CIMMYT) that were genotyped as part of the in Basic Research to Enable Agriculture Development (BREAD) project, Chinese-NAM (CN-NAM) population and the Goodman association panel^39^. Total genomic DNA was isolated from etiolated seedlings or leaf punches of all inbred lines using Qiagen Kits. The reduced representation libraries were constructed and sequenced following the GBS protocol^16^. DNA samples were digested with the restriction enzyme *Ape*KI, and then sequenced in on the Illumina Genome Analyzer or HiSeq 2000. Multiplexing of 96 or 384 samples was used on each flow cell lane.

Raw sequence reads were processed by GBS reference pipeline in TASSEL version 3.0 (refs 27, 28). In this pipeline, Illumina reads are trimmed to 64 bp. Identical 64-bp reads are considered to be a GBS tag (Supplementary Fig. 1). The tags with a minimum read count of 20 were used for genotyping. These tags were aligned to 4.38 million unique positions in B73 reference and covered ∼12% maize genome. Population genetic-based SNP filters were applied to filter putative SNPs. Genotypes were called using likelihood ratio test on potential genotypes^27^. The missing genotypes were imputed using an algorithm searching closest neighbour in a window, allowing for 5% mismatch. About 10% of genotypes were unimputed, since the requirements were not met. For any two inbred lines in the data set, ∼85% SNPs are nonmissing in both. About 1% SNPs are both missing in the two lines. The details of genotyping and imputation can be seen in a previous study^29^.

### Genetic mapping of GBS tags

Both GWAS and joint linkage mapping^30^ approaches were used to map GBS tags. The presence and absence of each tag was treated as a trait to be mapped on the anchor map of 681,257 SNPs (Supplementary Fig. 2). In the GWAS mapping, all of maize inbred lines were used. A binomial test was applied to detect the significant associations with SNPs. In the binomial distribution *X*∼B (*n*, *p*), where *n* is the intersection count of a tag and a SNP, *p* is the MAF of a SNP. A tag mapped to a SNP would be nonrandomly linked to one allele and generate a low *P* value. A value of 1E−6 was set as the threshold of GWAS. The position of a SNP with lowest *P* value was taken as the position of the tag. The joint linkage mapping was conducted in 5,000 NAM recombinant inbred lines. There were three steps in this mapping approach. First, the same binomial test was applied in each family to find the NAM families in which the tag was co-segregated with a SNP (*P* value <0.05). The SNP with the lowest *P* value represents the position of the tag. Second, we grouped the segregating families based on the chromosome where the tag was mapped. Third, the group with most segregating families was used to remap the tag to a higher resolution (*P* value <0.05).

It should be noted that mapping 26-M tags on nearly 700-k SNPs is extremely computationally expensive; therefore,we used several tactics to speed up the process. (1) Tags with a minimum count of 30 were used for mapping; (2) the population structure was not controlled for in GWAS mapping, which was a balance choice between speed and accuracy; (3) since the ‘trait’ and SNPs are all binary, they were compressed into bit sets for much higher speed by bit operation. These tactics reduced the computation time down to ∼27,000 central processing unit (CPU) hours, which became feasible while using clusters.

### Model training and prediction of mapping accuracy

ML models were trained to predict and select most accurately mapped tags from the initial mapping results. We aligned the 26-M tags to B73 reference genome using Bowtie2 with very-sensitive-local option^40^. The tags with only one hit, which are also a perfect match to the reference, were called UABTs. A total of 30,000 UABTs were included in the training data set. The distance between physical position and genetic position of a tag was taken as the dependent variable. Positions were transformed with an equation of pos=chromosome × 1E9+pos. A total of 16 attributes (Supplementary Table 1) were selected mostly based on biological considerations. The values of these attributes were normalized by box-cox transformation. Using Waikato Environment for Knowledge Analysis^32^, ML models (for example, decision tree, support vector machine and association rule) were tested (Supplementary Table 2). M5Rules had the best performance on the basis of the *r*^2^ and the mean error between predicted distance and observed distance (Supplementary Fig. 4). Since some tags were mapped only by GWAS or joint linkage mapping, some tags were mapped by both; we trained three M5Rules models, M5Rules_G, M5Rules_J and M5Rules_GJ, for the three classes of tags. Then, accurately mapped tags were to be selected on the basis of prediction accuracy (Supplementary Table 8). The 4.4-M mapped tags are available at http://www.panzea.org/dynamic/derivative_data/Lu_*etal*_2015_NatCommun_panGenomeAnchors20150219.txt.gz. To make the genetic mapping of GBS tags and ML filtering more useful for pan-genome projects of other species, this combined approach is available as a pipeline called Pan-genome Atlas (PanA). The document can be found at https://bitbucket.org/tasseladmin/tassel-5-source/wiki/Home.

### Population structure and genetic mapping accuracy

A principle component analysis was performed in all of the maize inbreds using 5,000 randomly chosen SNPs. The first three principle components (PCs), which explained 31% of total variance, were arbitrarily chosen to represent the population structure. The Pearson’s *r* was calculated between the 30,000 UABTs and the first three PCs. The values of *r* were used to as surrogates to indicate how much the presence and absence of an UABT was influenced by the population structure. The correlation between mapping accuracy (distance between physical position and genetic position of an UABT) and those surrogates of the population structure were calculated.

### Genetic diversity and genetic mapping performance

Two populations were selected from NAM and the Ames association panel. The population size was 400 for each. The selected NAM population comes from two NAM families, including B73XB97 and B73XOH7B. The selected Ames population was randomly chosen from Ames association panel, which were supposed to have much higher genetic diversity than in NAM. A total of 500,000 UABTs were genetically mapped in each population. Mapping results from 30,000 UABTs were used in ML training and prediction. The genetic mapping and ML modelling were performed in PanA.

### Assessment of sequence alignment accuracy in maize

To obtain a general picture of alignment accuracy in maize, 95 diverse maize inbreds that were selected perform the alignment accuracy evaluation. These samples were digested by the restriction enzyme *Ape*KI to guarantee that the sequences would overlap with 4.4-M mapped tags. The digested samples were sequenced using Illumina MiSeq 2 × 250 bp sequencing. Using the Smith–Waterman algorithm, the paired reads that had overlap were contiged together if the overlap was longer than 20 bp and the identity was greater than 90%. In this way, fragments with various length were generated and aligned to the B73 reference genome using Bowtie2 with very-sensitive-local option without imposing a mapping quality threshold. Those fragments whose first 64 bp could be found in 4.4-M anchors were used to compare their genetic position and physical position (Supplementary Fig. 10). If the physical position was not within the 10-Mb region of genetic position, the alignment was arbitrarily considered to be incorrect.

### Identifying PAV tags

The 4.4-M mapped tags were aligned to the 10-Mb region of B73 reference around their genetic positions using Bowtie2 with very-sensitive-local option. For each tag, if there was not any alignment found, the tag would be considered as a PAV tag. Owing to the fact that partially methylation-sensitive enzyme *Ape*KI is used in GBS and differential methylation exists across the genome, to calculate the distribution of PAV tags along the chromosomes, the number of PAV tags were normalized by the number of B73 tags in 1-Mb windows.

### Validation of PAV tags by CML247 *de novo* assembly

We recently started sequencing and assembling maize inbred line CML247 as a pilot project for the maize pan-genome. The idea was to find an optimized approach for maize *de novo* assembly by testing different sequencing platforms and assembly algorithms. Then, this optimized approach can be replicated in many other diverse maize inbred lines. Since maize has a complex genome, which challenges high-quality assembly, the 4.4-M sequence anchors are used as a quality control for these assemblies (Supplementary Fig. 13). So far, the NRGENE approach ( http://www.denovomagic.com) works well. Two paired-end PCR-free libraries, including 500 and 800 bp insert sizes, and three mate pair libraries including 3–5, 5–7 and 8–10 kb insert sizes, were sequenced to 130 × of CML247 genome using Illumina HiSeq 2500 and MiSeq. The N50 of NRGEGE CML247 assembly is 312 kb. The raw sequence data are available at http://www.panzea.org/db/feedback/CML247.

The 4.4-M sequencing anchors, including PAV tags, were aligned to NRGENE CML247 assembly using Bowtie2. A total of 200 high-quality scaffolds were used for PAV tags’ validation. The total length is 201 Mb. The minimum length of these scaffolds is 72 kb. In these scaffolds, >95% of aligned sequence anchors on each scaffold were from the same genomic region (Supplementary Fig. 13). The CML247 scaffolds were aligned to the B73 reference to find their orthologous regions. Then, each scaffold was aligned to its orthologous region using BLAST^41^ with default parameters, in which the word size is 11 bp. Since the short word size led to large number of small and spurious alignments, we used the following strategy to keep only orthologous alignments. For each scaffold and its B73 orthologue, 10 alignments with the highest score were selected as backbone alignments, which were alignments from large fragments and showed synteny between B73 and CML247. Any alignments crossed these 10 backbone alignments were removed (Supplementary Fig. 14). If a PAV tag on scaffolds did not have overlap with those aligned fragments on scaffolds, it would be considered as a valid PAV tag.

### GWAS on phenotypic traits

All of the 681,257 GBS SNPs were used in the GWAS. Phenotypic data of Ames association panel (2,951 inbred lines) and Goodman association panel (282 inbred lines) were collected from multiple locations over 3 years. A total of 2,661 inbred lines have available data of four complex traits, including days to silk, days to anthesis, plant height and ear height. GWAS analysis of these traits were performed on the basis of compressed mixed linear model^42^.

## Additional information

**How to cite this article:** Lu, F. *et al.* High-resolution genetic mapping of maize pan-genome sequence anchors. *Nat. Commun.* 6:6914 doi: 10.1038/ncomms7914 (2015).

## Supplementary Material

Supplementary Figures, Supplementary Tables and Supplementary References Supplementary Figures 1-18, Supplementary Tables 1-9 and Supplementary References

Supplementary Data 1 Best linear unbiased prediction value of four complex traits

Supplementary Data 2 GWAS results of four complex traits

Supplementary Data 3 Maize inbred lines used in genetic mapping of GBS tags

## Figures and Tables

**Figure 1:**
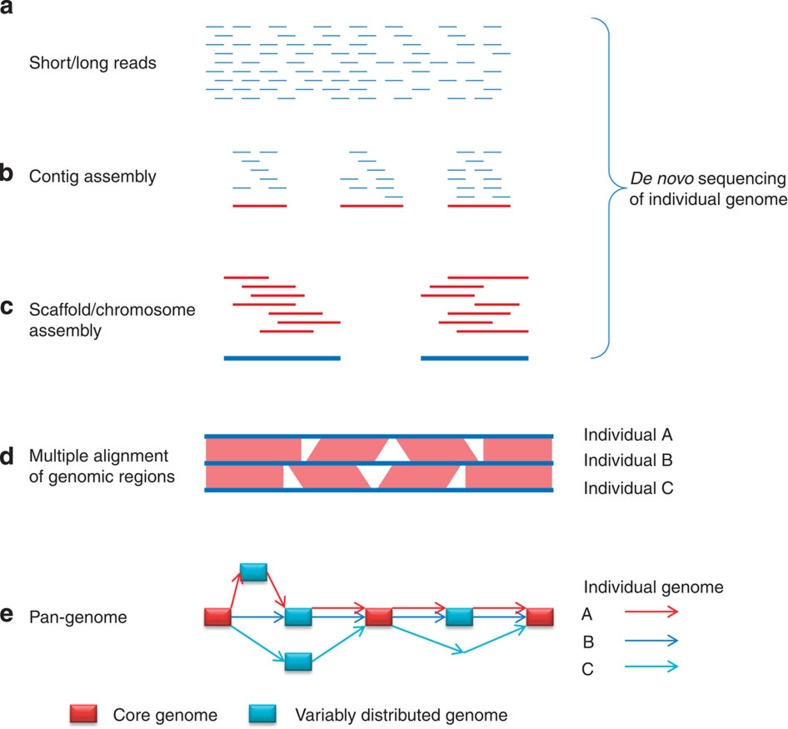
A framework to construct the pan-genome on the basis of *de novo* genome assemblies. (**a**) Individual genome is randomly sheared and sequenced using either short or long read sequencing technologies. (**b**) Contig assembly using reads. The contigs are usually generated with k-mers using *de Bruijn* graph-based algorithms. (**c**) Scaffold/chromosome assembly on the basis of contigs. (**d**) Identifying structural variations by sequence alignment. (**e**) The graph of the pan-genome. The rectangles represent genomic sequence. Red rectangles are sequences from the core genome, in which sequences are present in all individuals. Blue rectangles are sequences from the variable distributed genome, which show structural variations. Individual genomes are represented by these rectangles connected with arrows.
