# Supplementary material for: High-resolution genetic mapping of maize pan-genome sequence anchors
Source: Nat Commun. 2015 Apr 16;6:6914. doi: 10.1038/ncomms7914 (PMC4411285; doi:10.1038/ncomms7914)
Supplement: Supplementary Figures, Supplementary Tables and Supplementary References — Supplementary Figures 1-18, Supplementary Tables 1-9 and Supplementary References [file ncomms7914-s1.pdf]

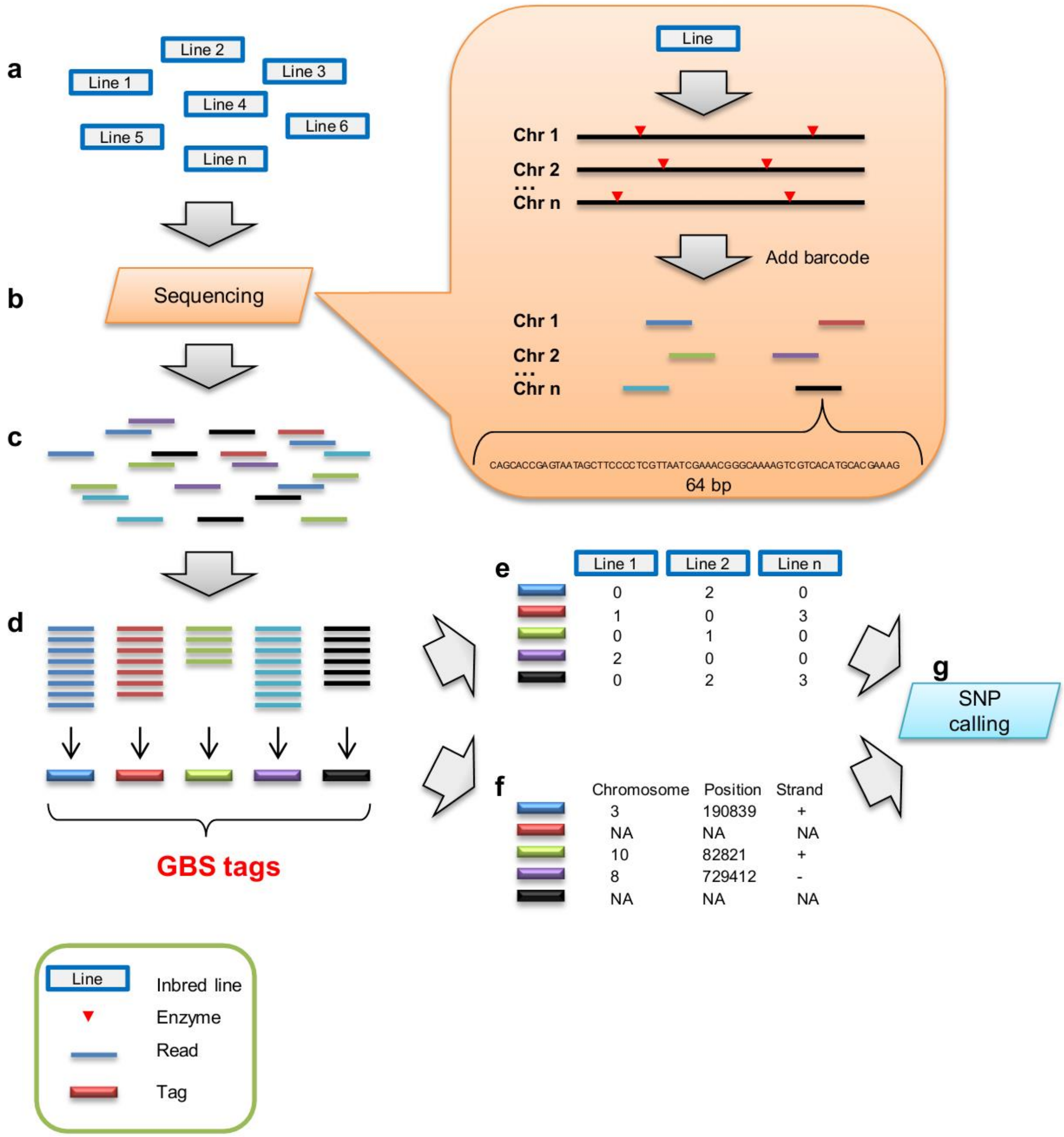


**Supplementary Figure 1** Genotyping by Sequencing (GBS) pipeline used in this study to genotype maize inbred lines. The 14,129 maize inbred lines were processed following GBS experimental design[1] and bioinformatics[2]. (**a**) Samples of maize inbred lines. (**b**) is the procedures in sequencing of GBS. Samples are digested with the restriction enzyme *Ape*KI. Samples are then barcoded and sequenced on Illumina platforms. Reads are trimmed to 64 bp *in silico*. (**c**) A pool of reads from all samples. (**d**)Identical reads are considered as a tag. (**e**) Tag count distribution across all maize inbreds. (**f**) Physical positions from alignment are recorded for these tags. (**g**) Tag count distribution and tag physical positions are used for SNP calling.

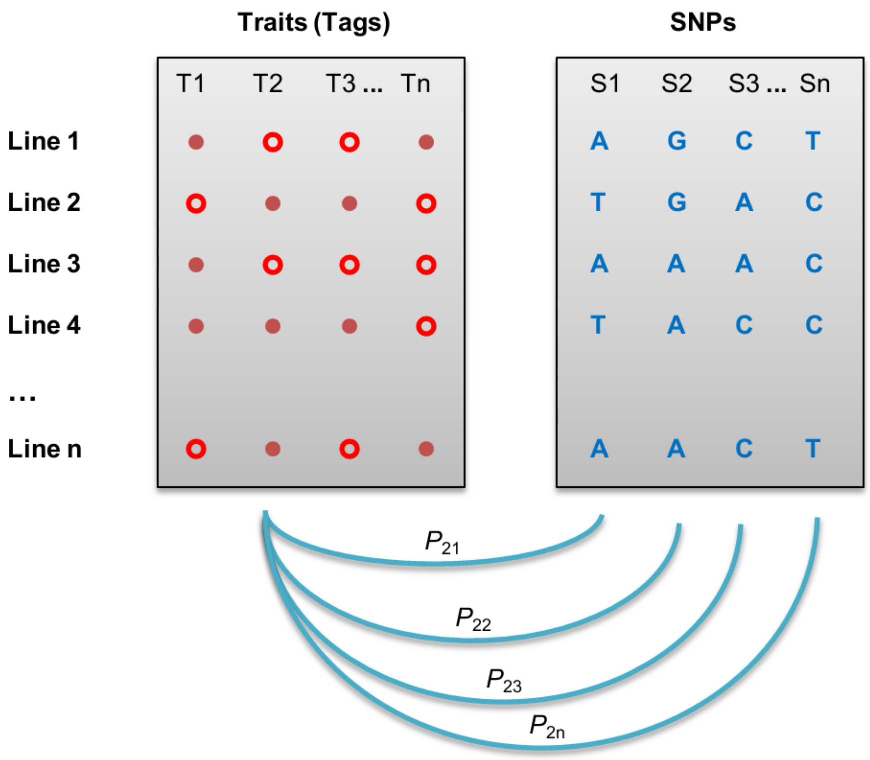


**Supplementary Figure 2** Genetic mapping of GBS tags. The presence (red dot) and absence (red circle) of a tag in inbreds is treated as a trait. Associations between each tag and all SNPs are tested. By comparing the *P*-values of associations, the position of the most significant SNP is taken as the genetic position of a tag.

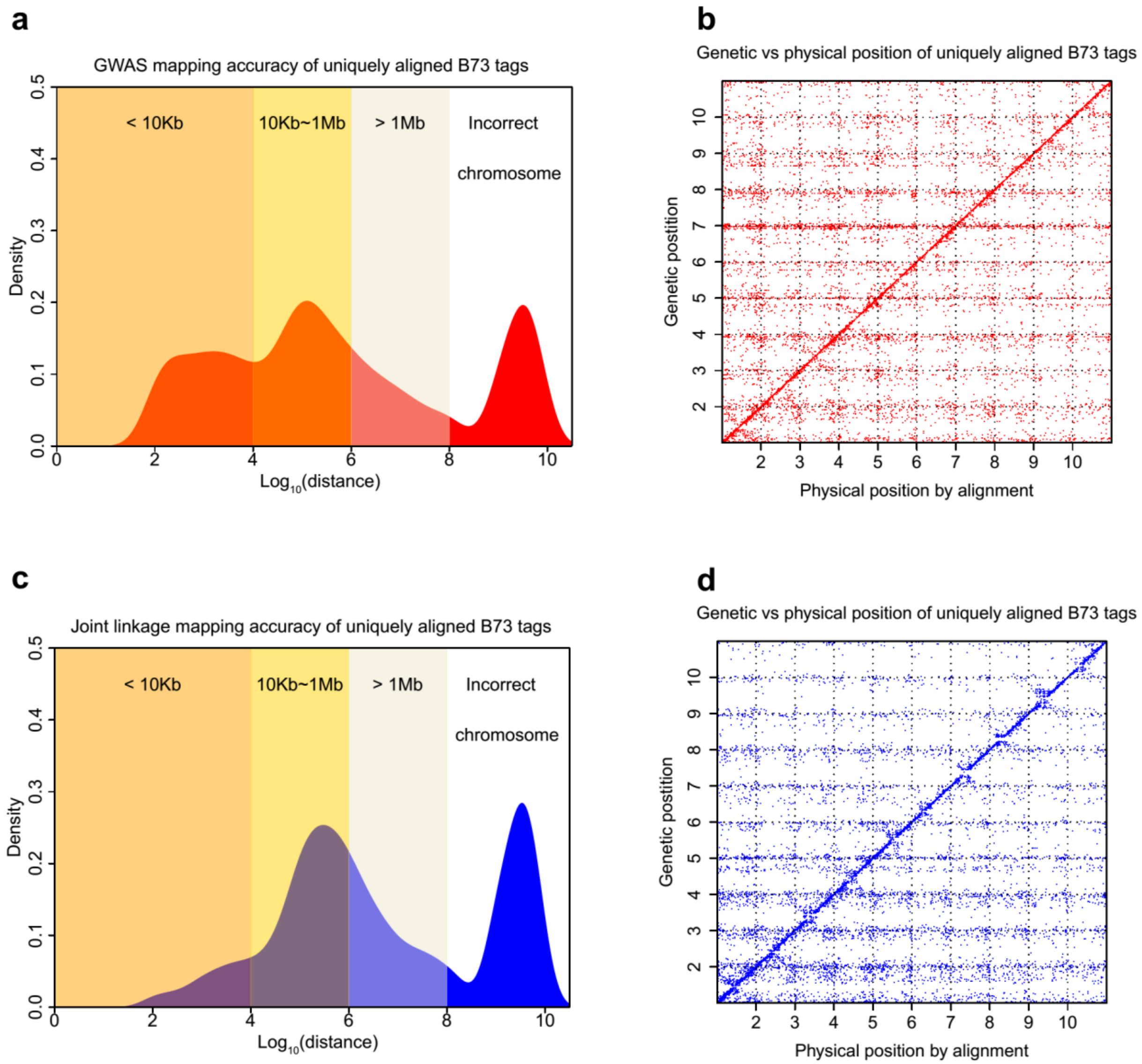


**Supplementary Figure 3** Initial tag mapping accuracy of GWAS and joint linkage mapping. A total of 20,000 randomly chosen UABTs were used as a quality control to evaluate the performance of genetic mapping. Subfigure (**a**) and (**b**) are results of GWAS. Subfigure (**c**) and (**d**) are results of joint linkage mapping. UABTs were used to evaluate the performance of genetic mapping. (**a, c**) shows the distribution of distance between genetic position and physical position (alignment position) of UABTs. Positions are transformed with an equation of pos = chromosome × 1E9 +pos. (**b,d**) are the scatter plots of genetic position against physical position of UABTs. The x and y axis are maize chromosomes.

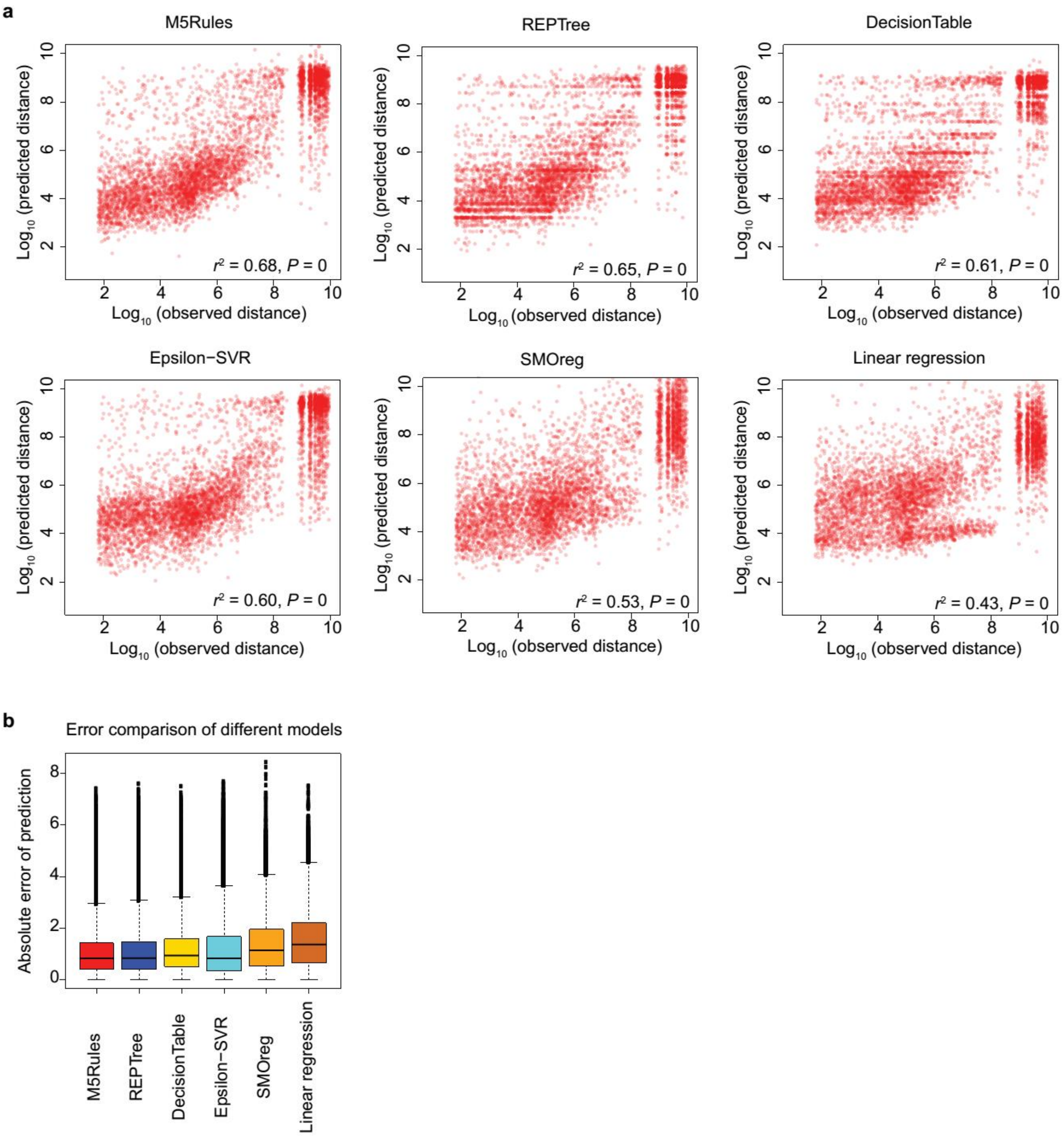


**Supplementary Figure 4** Performance comparison of multiple ML algorithms. The distance between physical position and genetic position of UABT was used as the dependent variable in these models. Six algorithms were tested for their performance using 30,000 UABTs. The Pearson's correlation coefficient ($r$) and mean error between observed distance and predicted distance were used as metrics to evaluate their performance. **(a)** are scatter plots of prediction and observation. The $r^2$ are labeled in each scatter plot. **(b)** are the error distributions. It is clear that M5Rules outperformed other five models.

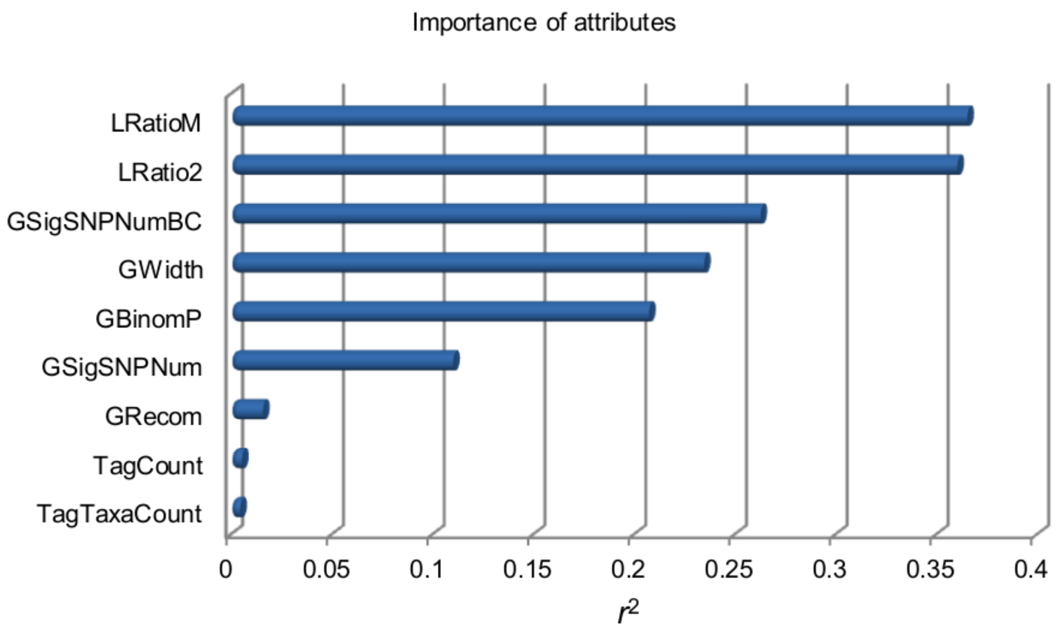


**Supplementary Figure 5** Importance of attributes used in M5Rules_G model. Attributes of 30,000 UABTs were collected to estimate the importance of attributes. Each attribute was used as a single variable in linear regression. The dependent variable is the distance between GWAS mapping position and observed position of UABTs. The $r^2$ is on the x-axis.

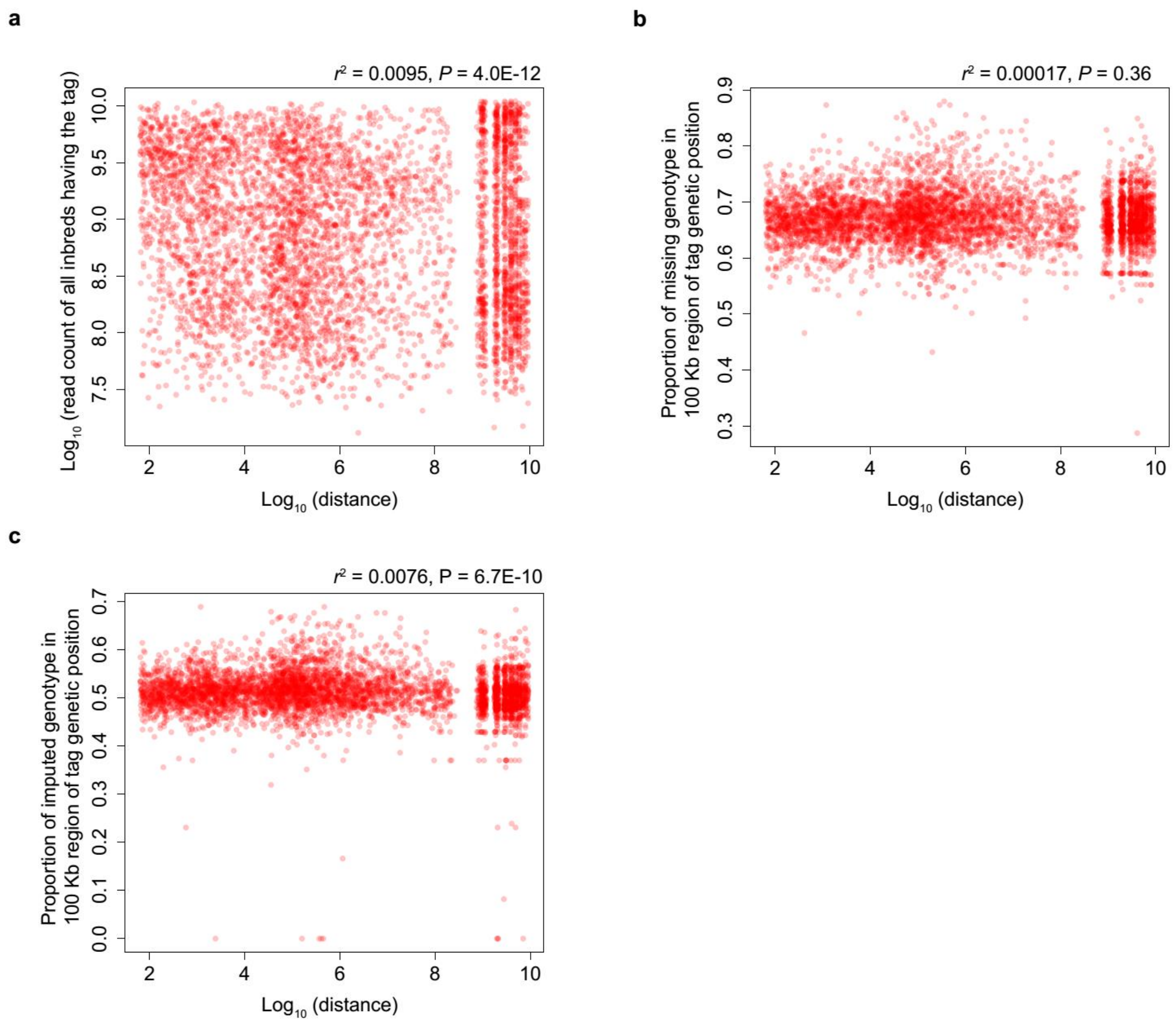


**Supplementary Figure 6** Effects of potential factors affecting genetic mapping accuracy. The distance between physical position and genetic position of 30,000 UABTs was used to calculate the $r^2$ with these potential factors. **(a)** The correlation between sequence depth of all inbred lines having the tag and genetic mapping accuracy. **(b)** The correlation between proportion of missing genotype of 100 kb window around tag genetic position and mapping accuracy. The genome was divided into 100 kb windows. The proportion of missing genotype was calculated across all 14,129 inbreds based on the unimputed genotype. The Pearson's *r* was calculated between the mapping accuracy and the missing value of the window where the tag was mapped. **(c)** The correlation between proportion of imputed genotype of 100 kb window around tag genetic position and mapping accuracy. The genome was divided into 100 kb windows. The proportion of imputed genotype was calculated across all 14,129 inbreds based on the comparison between imputed and unimputed genotypes. The Pearson's *r* was calculated between the mapping accuracy and the proportion of imputed data of the window where the tag was mapped.

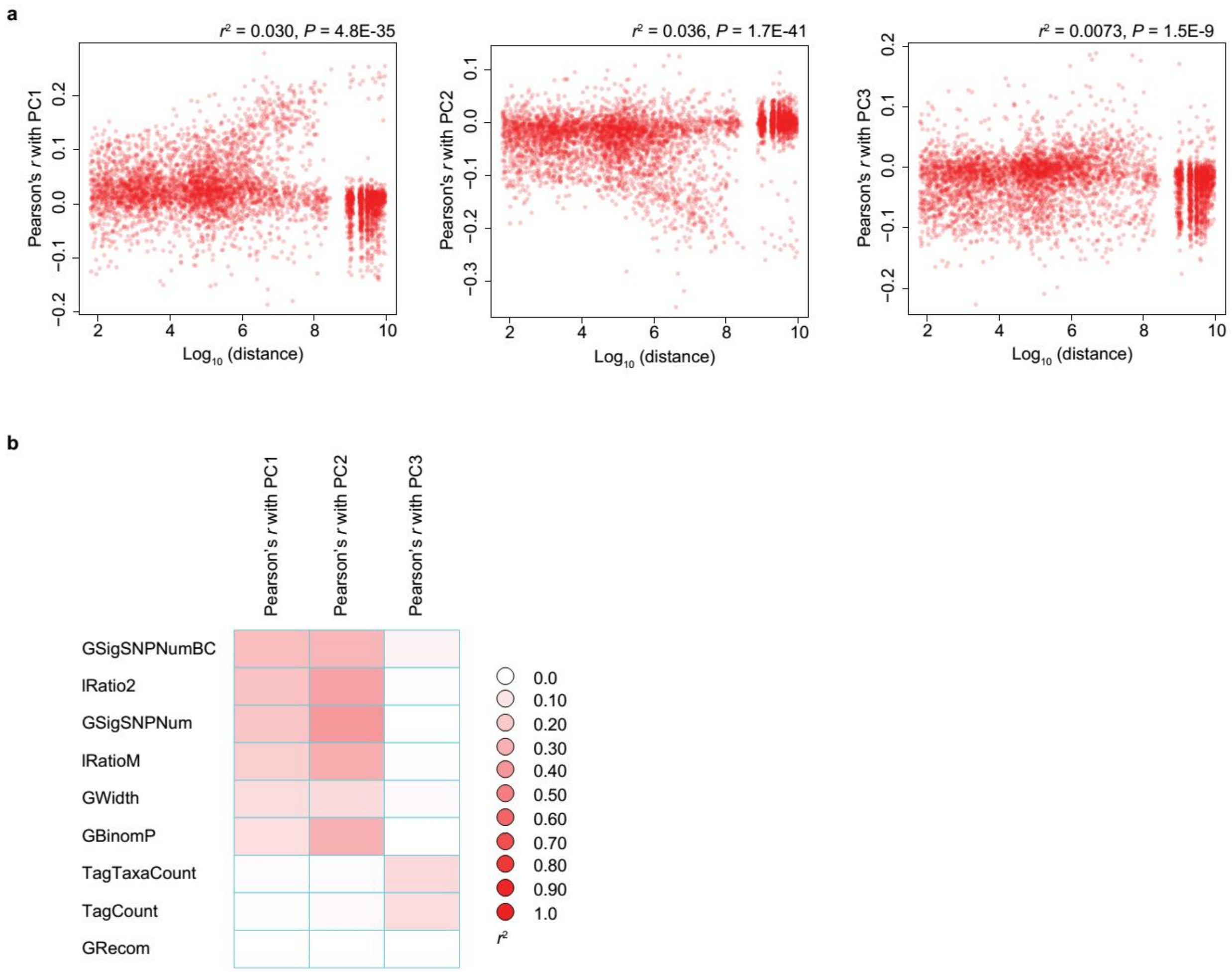


**Supplementary Figure 7** Population structure affects genetic mapping accuracy but its variance is captured by attributes in ML models. The distance between physical position and genetic position of 30,000 UABTs was calculated as mapping accuracy. To evaluate how population structure affects mapping result, a principle component analysis (PCA) was conducted in all 14,129 inbreds. The first three principle components (PCs) explained 20%, 7% and 4% of total variance. The Pearson's *r* was calculated between the first three PCs and the presence and absence of each UABT across all inbreds. The value of *r* served as a surrogate to indicate how much the presence and absence of the UABT was affected by population structure. **(a)** The correlation between population structure and mapping accuracy. Although the accuracy was slightly affected by population structures, especially for the 1st and 2nd PCs, it was clear that the less accurately mapped tags had higher proportion of tags associated with population structure. **(b)** The correlation between population structure and attributes in ML model. The first two PCs, which explained 27% of total variance, were correlated with 6 major predictors in M5Rules_G model (**Supplementary Fig. 6**)

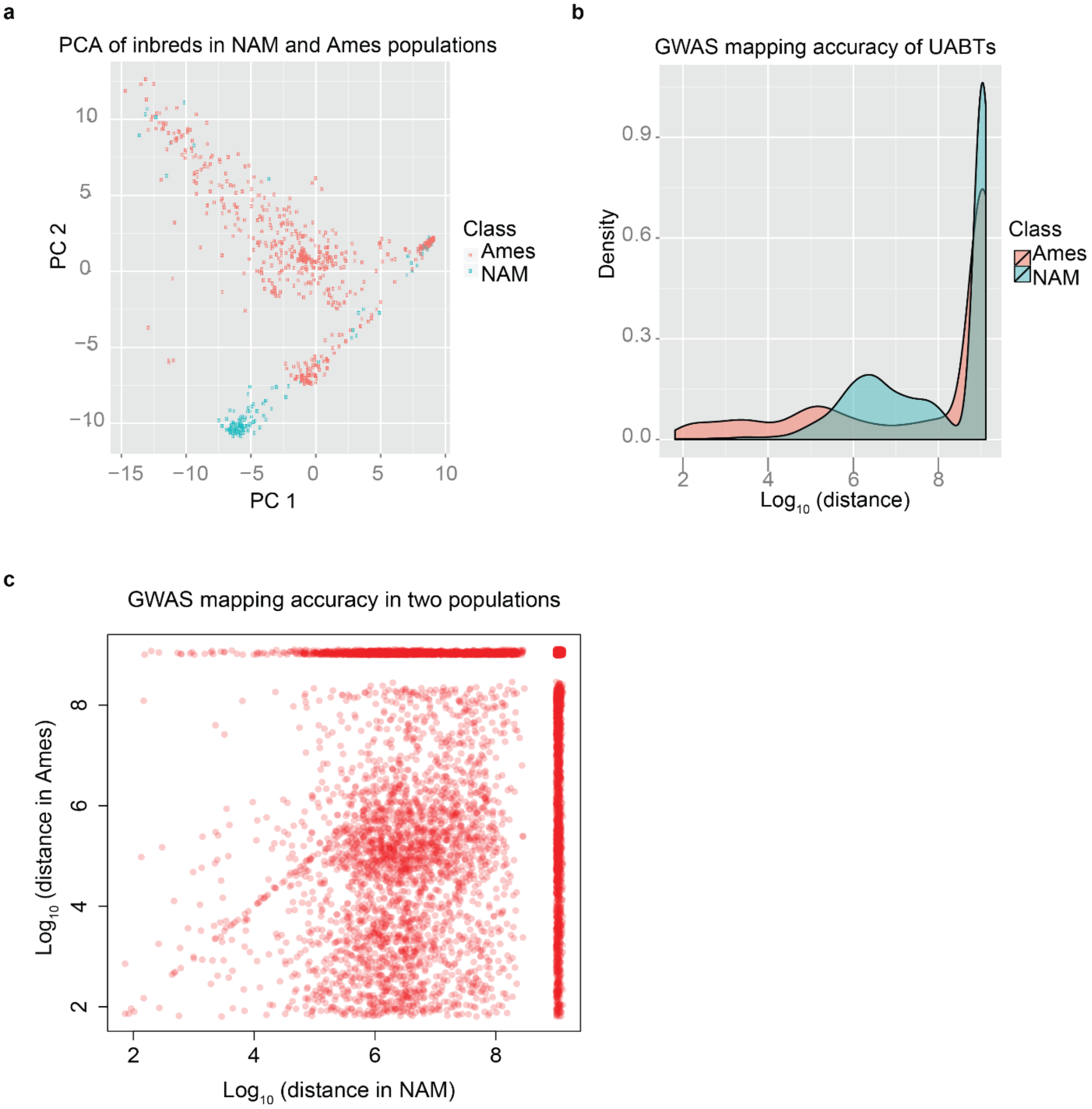


**Supplementary Figure 8** Comparison of genetic mapping performance in two populations of different diversity level. Two populations (each with n = 400) were used to conduct the comparison. One population was 2 families of NAM[3]. The other was randomly sampled from Ames association panel[4]. Tag GWAS mapping was performed in two populations with 500,000 UABTs, respectively. A number of 30,000 UABTs were used in ML training. **(a)** The principle component analysis in both populations. The first two principle components explained 20% and 13% of total variance. The inbreds selected from Ames showed much higher diversity. **(b)** The resolution distribution of mapping results from both populations. Genetic mapping in highly diverse population showed better resolution. **(c)** Scatter plot of mapping accuracy of tags mapped in both populations. Tag placement in high diverse population was more accurate.

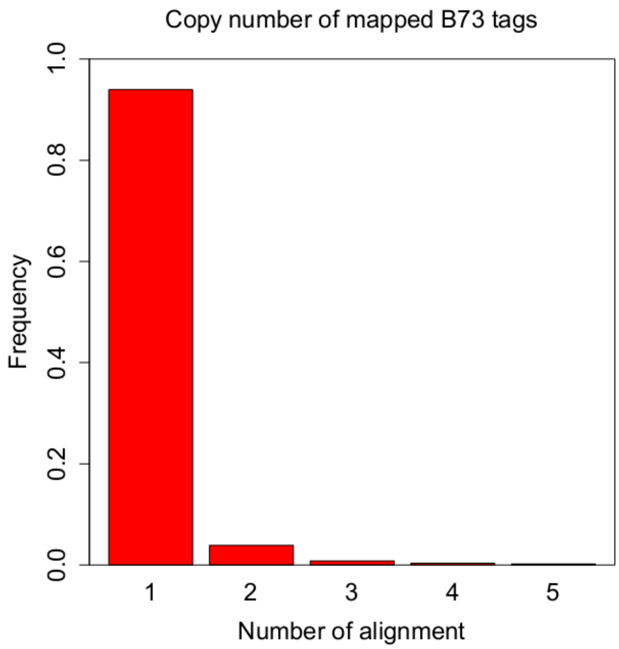


**Supplementary Figure 9** Copy number of mapped B73 tags. B73 tags (tags that are perfect match to the B73 reference genome) within 4.4M sequence anchors were aligned to the reference. Only the perfect alignment of B73 tags was counted here. About 94% B73 tags were single copy, suggesting that the 4.4M mapped tags were low copy sequences.

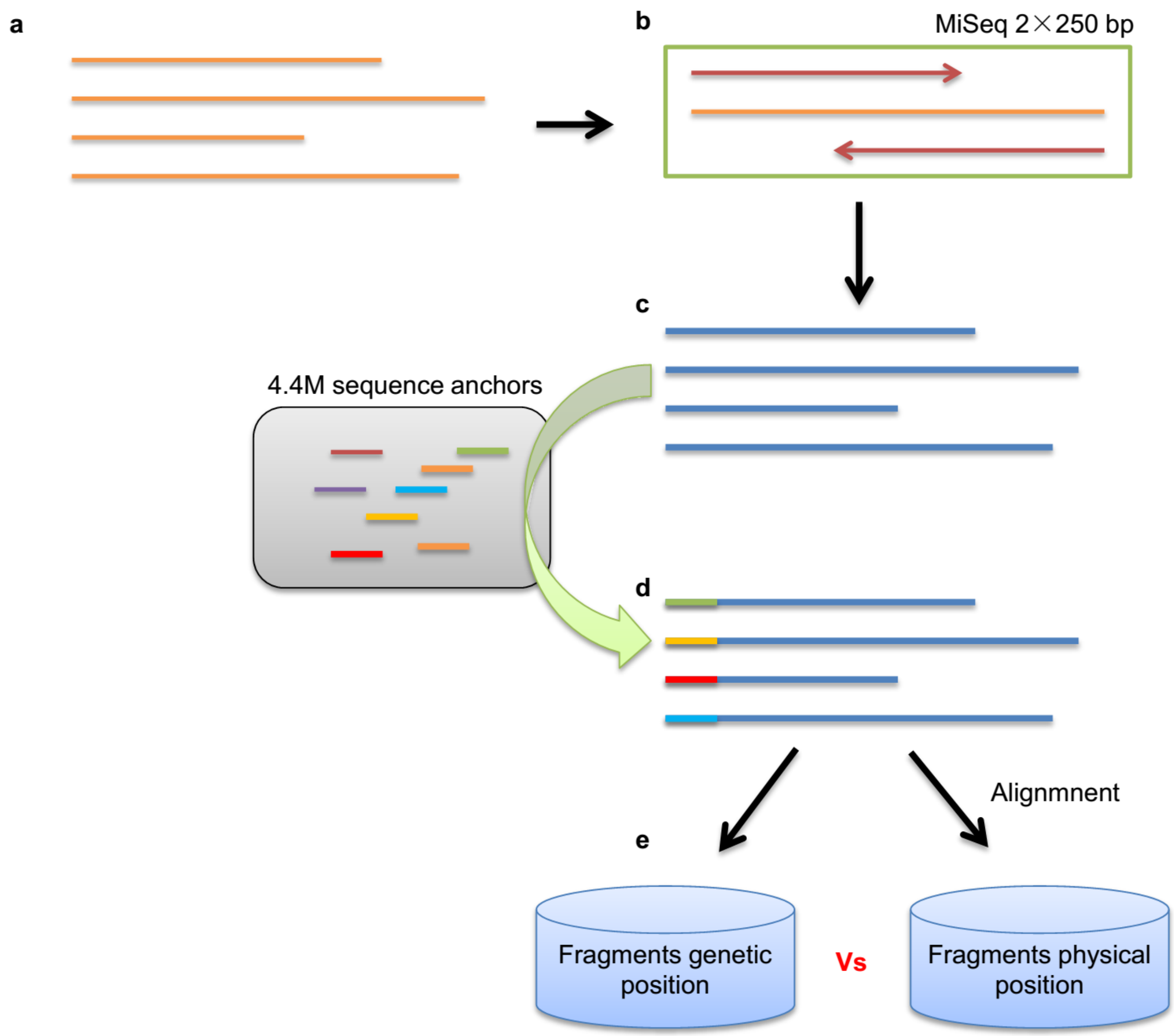


**Supplementary Figure 10** Evaluating alignment accuracy using paired end sequencing and 4.4M sequence anchors. A total of 95 highly diverse maize inbreds were digested with restriction enzyme *Ape*KI. These samples were then paired-end sequenced using MiSeq 2x250 bp protocol. Since the digested fragments had various sizes, most fragments whose size were less than 500 bp were able to be contiged together from reads of both ends. This graph shows the approach where used to evaluate alignment accuracy in maize. **(a)** DNA fragments were obtained after diverse maize samples were digested with *Ape*KI. **(b)** Samples were sequenced using MiSeq paired end sequencing. **(c)** Fragments with overlapped paired end reads were contiged together. **(d)** Looking for genetic positions of fragments from 4.4M sequence anchors. **(e)** Compare genetic position and physical position (alignment position) of fragments of various sizes.

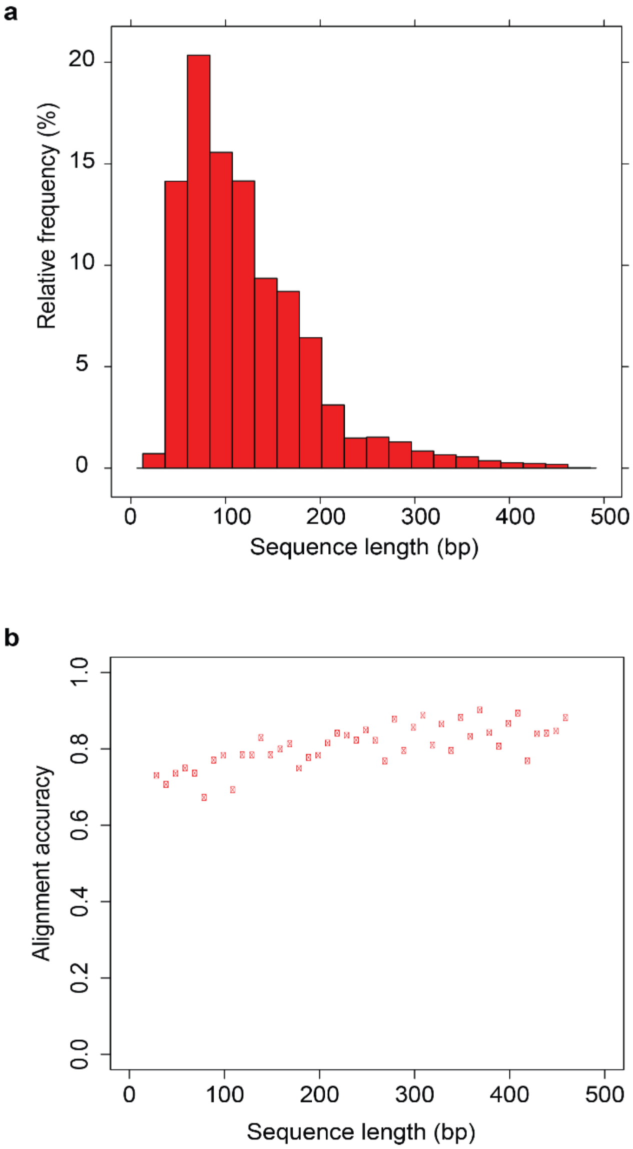


**Supplementary Figure 11** Agreement between physical position (alignment position) and genetic position of maize genomic sequences. Using the approach described in **Supplementary Fig. 12**, 5,281,392 unique reads were generated from 95 most diverse maize samples. A total of 3,101,793 unique reads were contiged together. **(a)** is the sequence length distribution of all the fragments, including contiged reads, forward and reverse reads. **(b)** is the alignment accuracy of fragments of different length. Fragments whose first 64 bp can be found from 4.4M sequence anchors were used to test the alignment accuracy. Assuming alignments whose physical position falling in 10 Mb region of genetic position were correct, we found the accuracy ranged from 70% to nearly 90% for fragments having length from 64 bp to 500 bp. It is worth noting that for those fragments of between 150 bp to 300 bp, which are the standard output sequence length of Illumina machines, the accuracy was only about 80%. This result exhibited the challenges of maize *de novo* genome assembly.

**a**

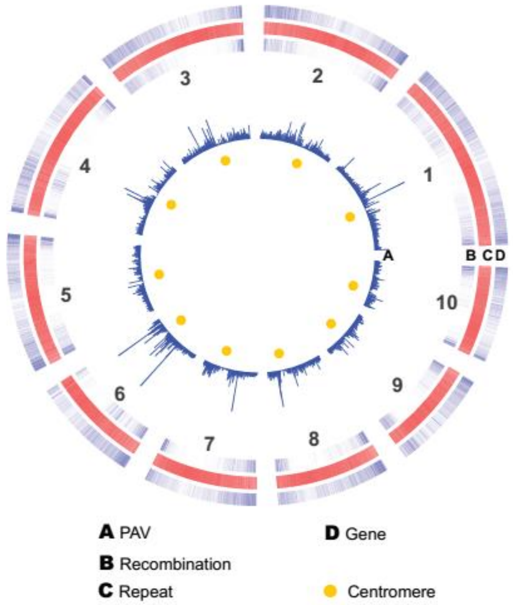


**b**

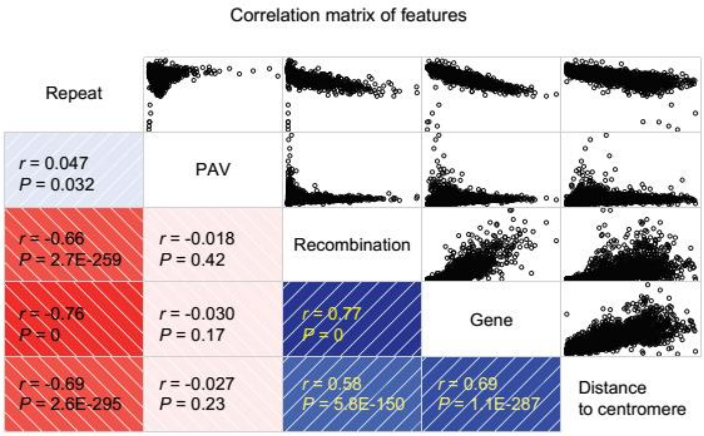


**Supplementary Figure 12** Distribution of PAV and biological features in maize genome. Recombination were calculated from the NAM population based on GBS markers in this study. **(a)** Distribution of PAVs and biological features in 1 Mb windows in maize genome. **(b)** Correlation matrix of PAV and biological features. The Pearson's *r* and *P*-values are labeled. The PAV was positively correlated with repeat density, but negatively correlated with recombination and gene distribution. However, the correlation between PAV distribution and gene distribution as well as recombination was not significant. This is due to the fact that GBS tags are more distributed in gene region where recombination events happen more often, because the restriction enzyme *Ape*KI used in GBS is a partially methylation sensitive enzyme. This diluted the correlation between PAV and other biological features.

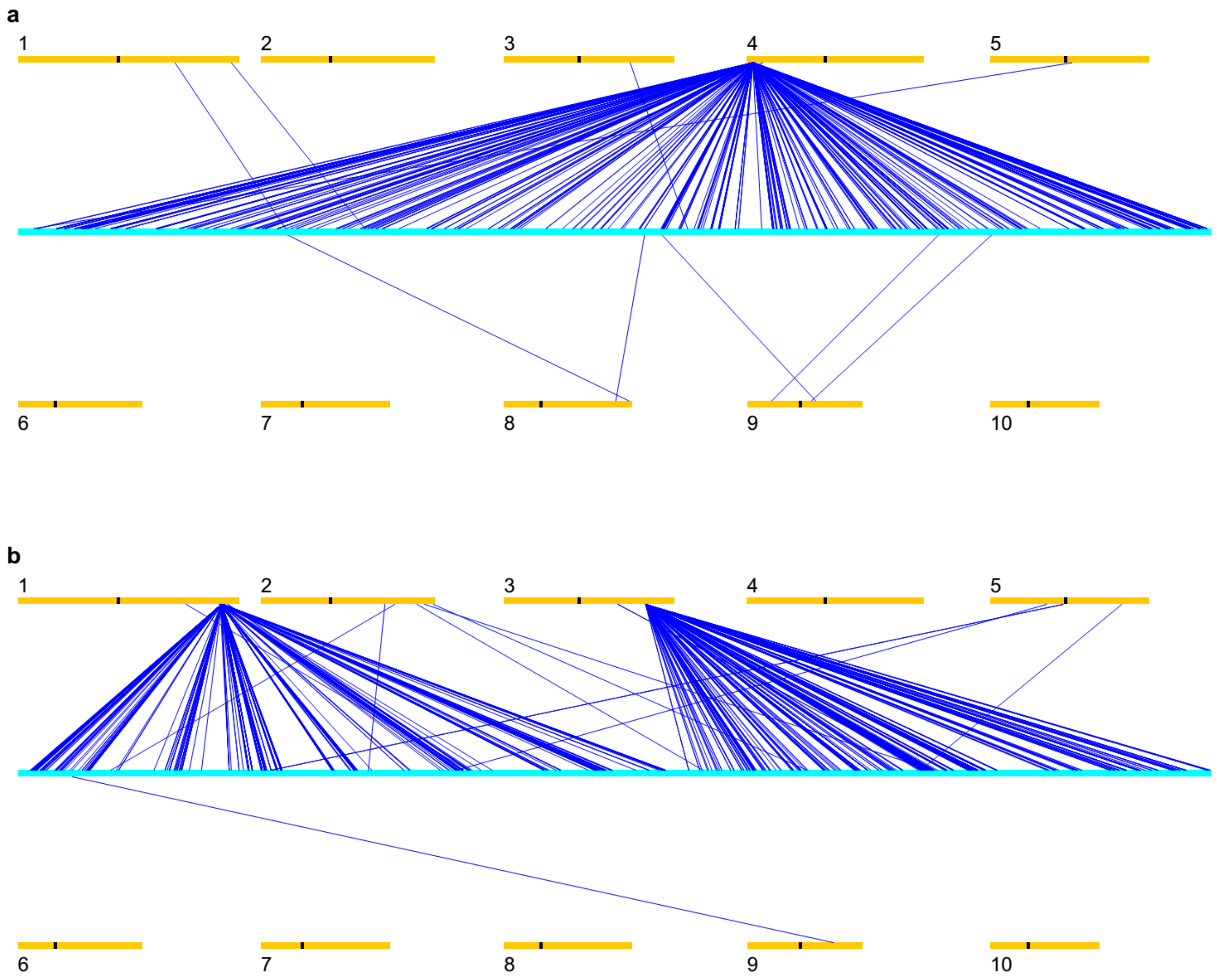


**Supplementary Figure 13** Evaluating quality of *de novo* genome assembly of maize inbred CML247 using 4.4M genetic anchors. To assess the genome assembly quality, the 4.4M sequence anchors were aligned to the scaffolds in the assembly. Only the anchors which showed unique and perfect match to the scaffolds were used for further evaluation. For a well assembled scaffold, the genetic positions of all the anchors on the scaffold are supposed to around the same genomic region. In contrast, for a poorly assembled scaffold, the anchors on one scaffold are usually clustered into multiple regions or scatter around all chromosomes. **(a)** is an example of well assembled scaffold. The cyan bar in the middle is the scaffold to be assessed. The orange bars at the top and bottom are ten chromosomes of maize. The blues lines showed the genetic positions of those anchors on the scaffold. The length of the scaffold is 923,871 bp. A total of 499 anchors (98.2%) pointed to the same genomic region. **(b)** is an example of incorrectly assembled scaffold. The length of this scaffold is 931,094 bp, with 769 anchors on it. It is clear that the scaffold resulted from two misplaced contigs.

a

b

c

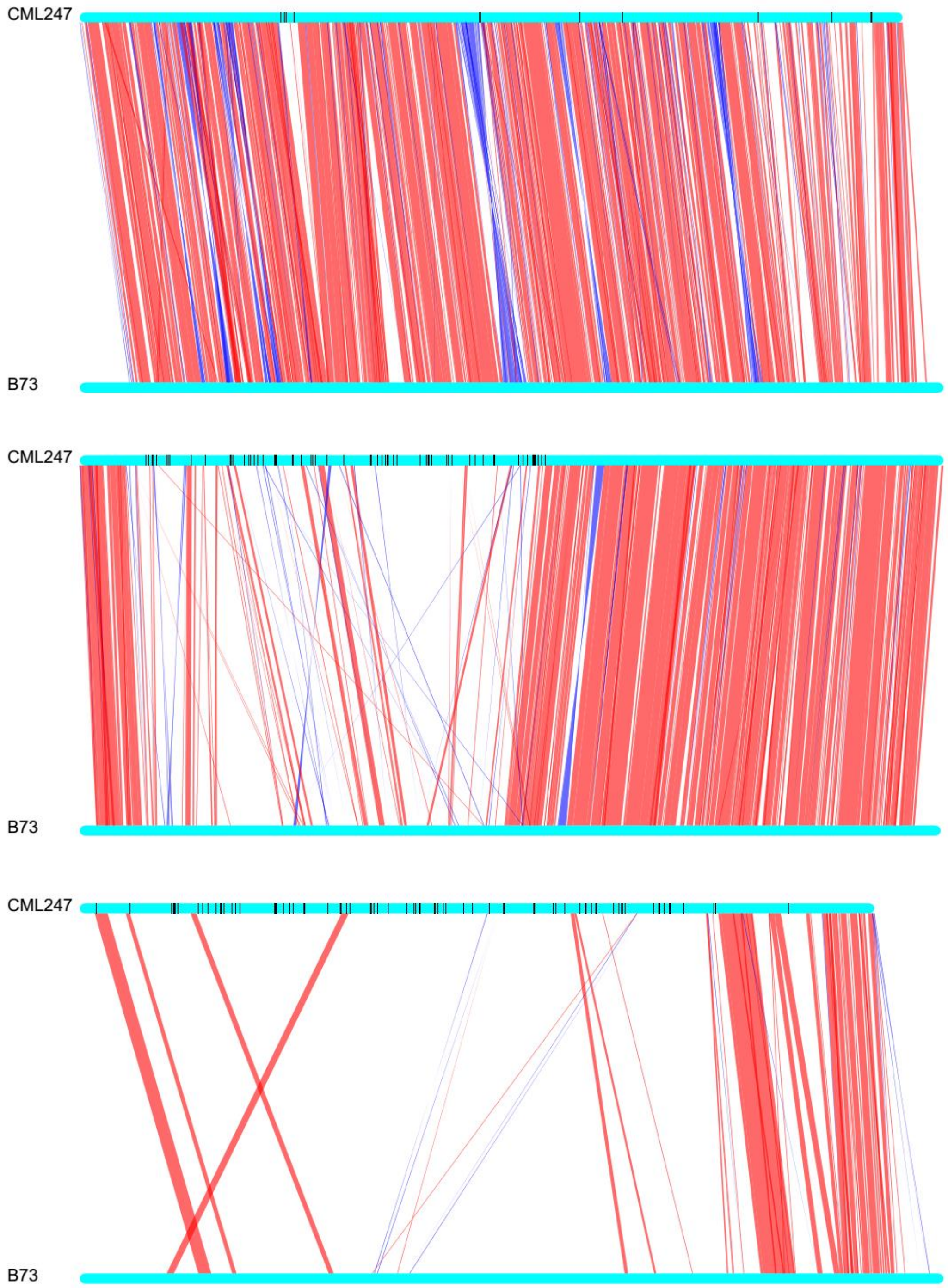


**Supplementary Figure 14** PAV tags are validated by alignment between orthologous regions of B73 reference and CML247. A total of 200 high quality CML247 scaffolds (total length = 201 Mb) were used to validate the PAVs tags identified in this study. For each scaffold, above 95% of sequence anchors were from the same genomic region (**Supplementary Fig. 15 a**). All the CML247 scaffolds were aligned to B73 reference genome to identify their orthologous regions. PAV tags were validated by examining orthologous alignments between B73 and CML247. (a) are alignments in a conserved region. The scaffold length is 2,895,935 bp. The cyan bars at the top and bottom are CML247 scaffold and its

orthologous region in B73, respectively. The red and blue between B73 and CMl247 are forward and reverse alignments, respectively. The black lines represent PAV tags identified in this study. (b) are alignments in a diverse region. The scaffold length is 2,156,505 bp. (c) are alignments in a highly diverse region. The scaffold length is 1,012,073 bp. Analyzing all of the alignments in 200 scaffolds, we found 89% of PAV tags on CML247 didn't show alignment in their orthologous B73 regions. The invalidated PAVs were due to the low sensitivity while aligning short 64 bp sequence anchors to the B73 reference during the process of PAV discovery.

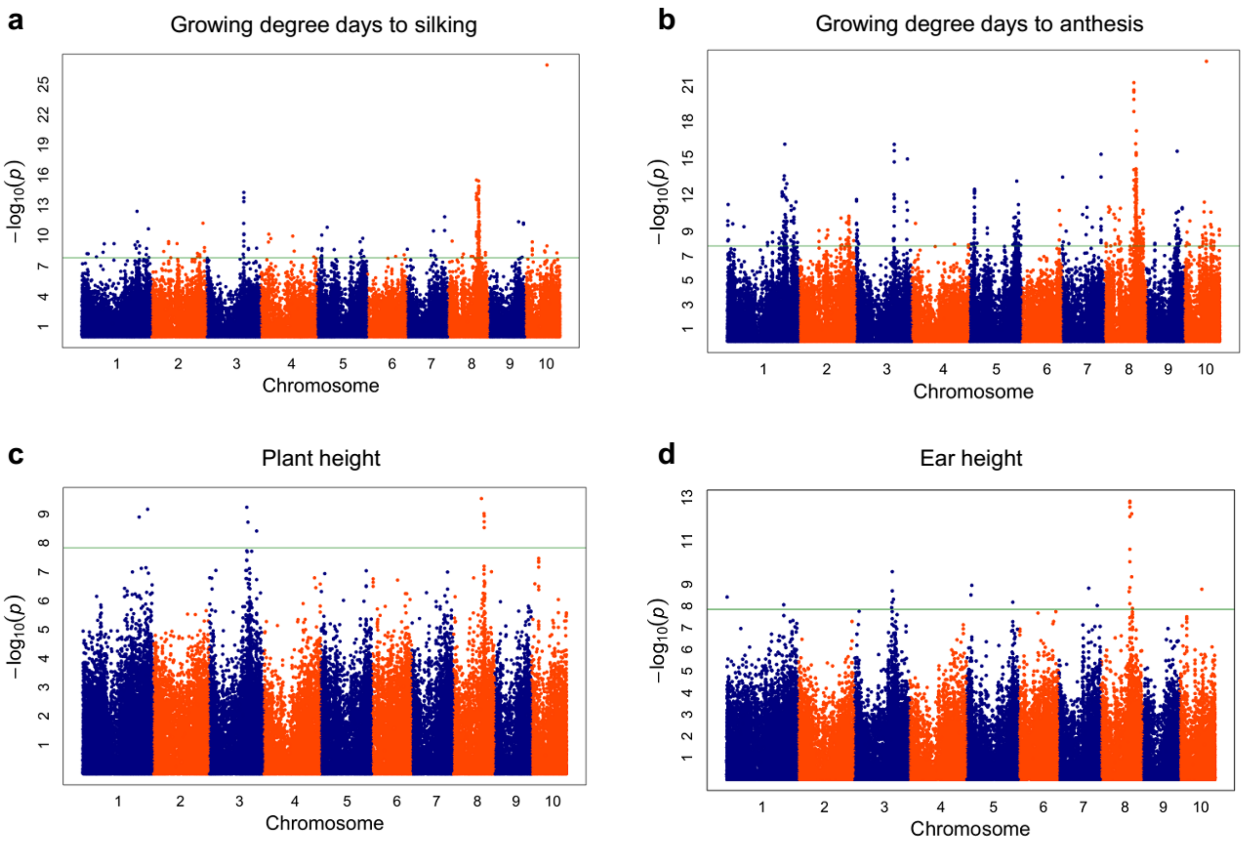


**Supplementary Figure 15** Manhattan plot of GWAS result of four complex traits. GWAS were performed in 2,661 diverse maize inbred lines using 700K SNPs. The red line indicates false discovery rate (FDR) threshold.

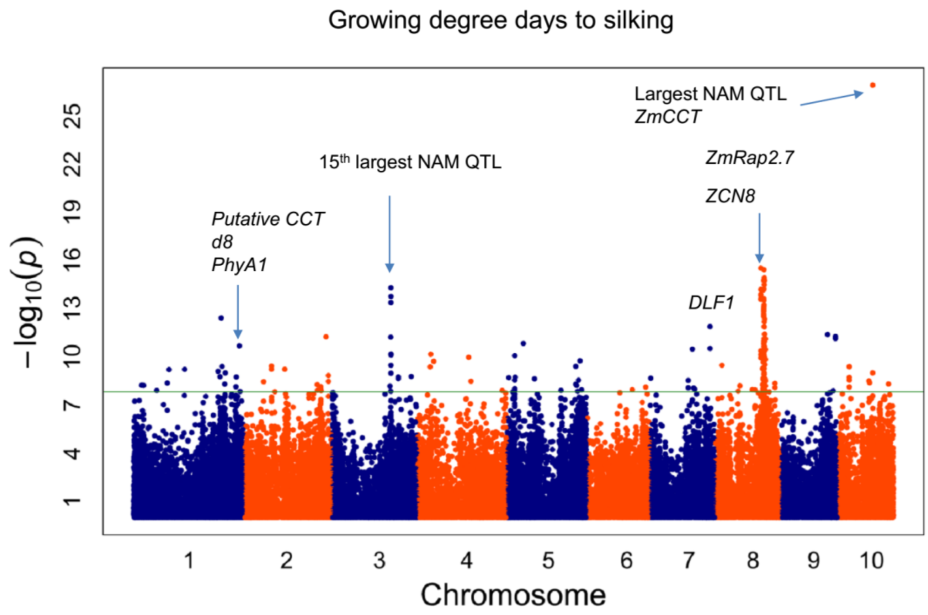


**Supplementary Figure 16** Quantitative trait loci (QTL) of maize flowering time trait are identified by GWAS. GWAS was performed for growing degree days using 700K SNPs across 2,661 diverse maize inbred lines. Many known QTLs involved in flowering time were close to those significant SNPs. These known QTLs are *ZmCCT*[5], *d8*[6], *PhyA1*[7], *ZmRap2.7*[8], *ZCN8*[9] and *DLF1*[10].

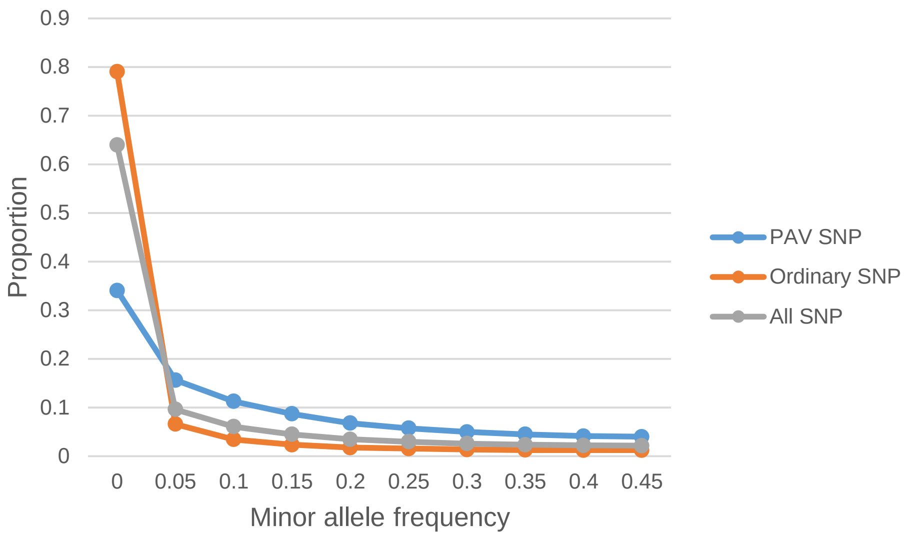


**Supplementary Figure 17** Site frequency spectrum (SFS) of PAV SNP and ordinary SNP. The 700K SNPs were used in this analysis. The minor allele frequency distribution of all the 700K SNPs serves as the null distribution of SFS in the population. The interval of minor allele frequency (MAF) is 0.05. PAV SNPs had a higher minor allele frequency (MAF) than ordinary SNPs.

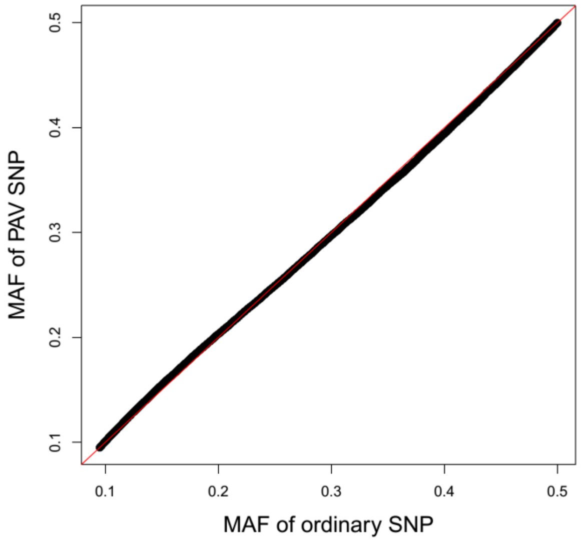


**Supplementary Figure 18** QQ-plot of MAF from both PAV SNPs and ordinary SNPs. After filtering out SNPs with MAF < 0.095, the PAV SNPs and ordinary SNPs had equal MAF distribution.

**Supplementary Table 1** Attributes used in ML model training

| Attribute | Description | Motives that attributes were chosen | Usage** |
|---|---|---|---|
| TagCount | Tag count | Associated with how repetitive the tag is. Repetitive tags are unlikely to be mapped | 1, 3 |
| TagTaxaCount | Number of taxa in which the tag is sequenced | Associated tag frequency and repetitiveness of the tag. Tag frequency affects power of association test | 1, 3 |
| G_Recom | Recombination rate of GWAS mapping position | Recombination rate affects mapping resolution | 1, 3 |
| G_BinomP | Binomial *P*-value of GWAS mapping | Significance of association between tag and SNP | 1, 3 |
| LRatio2 | Likelihood ratio of the best mapping chromosome vs the second best chromosome (GWAS) | Measure the spurious association due to population structure. True association should have high value of LRatio2 | 1, 3 |
| LRatioM | Likelihood ratio of the best mapping chromosome vs the median best chromosome (GWAS) | Measure the spurious association due to population structure. True association should have high value of LRatioM | 1, 3 |
| G_SigSNPNum | Number of SNPs which pass the threshold *P*-value (GWAS) | Associated with population structure and repetitiveness of tags | 1, 3 |
| G_SigSNPNumBC | Number of SNPs which pass the threshold *P*-value on the best chromosome (GWAS) | Associated with population structure and confidence of tag association test | 1, 3 |
| G_Width | Physical distance between the first significant SNP and the last significant SNP on the best chromosome (GWAS) | Associated with size of LD block. Mapping resolution may drop in large LD blocks. | 1, 3 |
| J_Recom | Recombination rate of joint linkage mapping position | Recombination rate affects mapping resolution | 2, 3 |
| J_BinomP | Binomial *P*-value of joint linkage mapping | Significance of association between tag and SNP | 2, 3 |
| J_SigSNPNumBC | Number of SNPs which pass the threshold *P*-value of joint linkage mapping on the best chromosome | Associated with population structure and confidence of tag association test | 2, 3 |
| FamilyNum | Number of NAM families in which the tag is mapped to the best chromosome | Confidence of tag association test of joint linkage mapping | 2, 3 |
| GJ_Distance | Physical distance between GWAS mapping position and joint linkage mapping position | Agreement between GWAS and joint linkage mapping | 3 |
| JDist* | Physical distance between observed position and joint linkage mapping position | Mapping accuracy | 2 |
| GDist* | Physical distance between observed position and GWAS mapping position | Mapping accuracy | 1,3 |

*are dependent variables
** shows in which model these attributes were used. Code 1, 2 and 3 represents M5Rules_G, M5Rules_J and M5Rules_GJ

**Supplementary Table 2** Machine learning algorithms evaluated for predicting tag mapping accuracy

| Algorithms | Type | Parameters in WEKA library[11] |
|---|---|---|
| M5Rules | Association rule | -M 50 |
| DecisionTable | Association rule | -X 1 -S "weka.attributesSelection.BestFirst -D 1 -N 5" |
| REPTree | Decision tree | -M 2 -V 0.001 -N 3 -S 1 -L -1 |
| Epsilon−SVR | Support vector machines | -S 3 -K 2 -D 3 -A5 0.0 -R 0.0 -N 0.5 -M 40.0 -C 1.0 -E 0.001 -P 0.1 -seed 1 |
| SMOReg | Support vector machines | -C 1.0 -N 0 -I "weka.classifiers.functions.supportVector.RegSMOImproved -L 0.001 -W 1 -P 1.0E-12 -T 0.001 -V" - K "weka.classiifiers.functions.supportVector.PolyKernel -C 250007 -E 1.0" |
| Linear regression | Regression | -S 0 -R 1.0E-8 |

**Supplementary Table 3** Rules and linear models of M5Rules_G

| Rule ID | Rule | Linear model |
|---|---|---|
| 1 | LRatio2 > 7.316<br>GSigSNPNum <= 32.046<br>GRecom > -3.078 | GDist = 1.2405 * TagCount + 0.8344 * TagTaxaCount - 2.16 * GRecom - 0.0143 * GBinomP - 0.09 * lRatio2 + 1.0973 * lRatioM - 0.0302 * GSigSNPNum + 0.4199 * GSigSNPNumBC + 0.0116 * GWidth - 3.0647 |
| 2 | 2.207 < LRatio2 < 4.046<br>TagCount > 8.417<br>GSigSNPNum <= 35.286<br>GSigSNPNumBC <= 3.802 | GDist = 1.9583 * TagCount + 1.3487 * TagTaxaCount - 2.5692 * GRecom - 0.0175 * GBinomP - 0.1157 * lRatio2 - 1.0189 * lRatioM - 0.0362 * GSigSNPNum + 0.4583 * GSigSNPNumBC + 2.0E-4 * GWidth - 4.0869 |
| 3 | LRatio2 > 3.671<br>TagCount <= 8.417<br>GSigSNPNumBC <= 2.961 | GDist = 0.5548 * TagCount + 0.4216 * TagTaxaCount - 3.1044 * GRecom - 0.0349 * GBinomP - 0.8577 * lRatio2 - 0.0101 * lRatioM - 0.0239 * GSigSNPNum - 0.2433 * GSigSNPNumBC + 0.0055 * GWidth + 4.562 |
| 4 | 1.382 < LRatio2 < 4.503<br>TagCount <= 8.453<br>1.823 < GSigSNPNumBC > 4.232 | GDist = -0.1003 * TagCount + 0.1266 * TagTaxaCount + 1.9791 * GRecom - 0.0349 * GBinomP - 1.1617 * lRatio2 - 0.21 * lRatioM + 0.0158 * GSigSNPNum - 0.7136 * GSigSNPNumBC + 0.0082 * GWidth + 2.1769 |
| 5 | LRatio2 > 2.207<br>TagCount > 8.417<br>GSigSNPNumBC > 2.607 | GDist = 2.5793 * TagCount + 1.7971 * TagTaxaCount - 1.0797 * GRecom - 0.0229 * GBinomP + 0.0026 * lRatio2 - 0.8016 * lRatioM - 0.1224 * GSigSNPNum + 1.3569 * GSigSNPNumBC + 0.0314 * GWidth - 1.3426 |
| 6 | 0.247 < LRatio2 <= 2.207<br>TagCount > 7.583<br>TagTaxaCount <= 13.902 | GDist = 0.0032 * TagCount + 0.0018 * TagTaxaCount + 1.3985 * GRecom + 0.022 * GBinomP - 0.0024 * lRatio2 - 1.2281 * lRatioM - 0.0732 * GSigSNPNum - 0.0048 * GSigSNPNumBC - 0.0063 * GWidth - 14.6559 |
| 7 | 0.247 < LRatio2 <= 0.936<br>TagTaxaCount <= 10.203 | GDist = 1.0599 * TagCount + 0.3777 * TagTaxaCount - 0.7967 * GRecom + 0.0703 * GBinomP - 3.0E-4 * lRatio2 - 1.9824 * lRatioM - 0.1089 * GSigSNPNum - 0.3515 * GSigSNPNumBC - 0.0 * GWidth - 8.0897 |
| 8 | LRatio2 <= 0.936<br>TagTaxaCount > 9.315<br>GRecom > -2.884<br>LRatioM <= 2.008 | GDist = -0.1007 * TagCount + 0.1178 * TagTaxaCount + 0.0216 * GRecom + 2.0E-4 * GBinomP - 0.0011 * lRatio2 + 0.015 * lRatioM - 0.0321 * GSigSNPNum - 3.0E-4 * GSigSNPNumBC - 0.0049 * GWidth - 9.3727 |
| 9 | LRatio2 <= 0.936 | GDist = 0.2309 * TagCount + 1.0E-4 * TagTaxaCount + 0.8688 * GRecom + 0.0101 * GBinomP - 4.0E-4 * lRatio2 + 0.6091 * lRatioM - 0.0494 * GSigSNPNum - 7.0E-4 * GSigSNPNumBC - 0.0058 * GWidth - 10.3943 |
| 10 | TagCount <= 8.765<br>1.167 < GSigSNPNumBC <= 4.232<br>GRecom > -3.109<br>GSigSNPNum > 22.694<br>LRatio2 > 5.34 | GDist = 0.192 * TagCount + 0.0176 * TagTaxaCount - 0.7755 * GRecom - 3.0E-4 * GBinomP - 0.2284 * lRatio2 - 0.0384 * lRatioM - 0.0849 * GSigSNPNum + 0.5877 * GSigSNPNumBC + 1.0E-4 * GWidth + 1.7937 |
| 11 | TagCount <= 8.823<br>1.167 < GSigSNPNumBC <= 3.706 | GDist = 0.9096 * TagCount + 0.543 * TagTaxaCount - 2.2969 * GRecom - 0.035 * GBinomP - 0.6234 * lRatio2 - 0.7126 * lRatioM - 0.0011 * GSigSNPNum + 0.134 * GSigSNPNumBC + 0.0268 * GWidth + 3.0989 |
| 12 | GSigSNPNumBC > 3.149<br>GSigSNPNum <= 34.461 | GDist = 0.2147 * TagCount + 0.0035 * TagTaxaCount - 2.7903 * GRecom - 1.0E-4 * GBinomP + 0.3192 * lRatio2 - 0.0222 * lRatioM - 0.0632 * GSigSNPNum + 0.6258 * GSigSNPNumBC + 0.0337 * GWidth - 5.0051 |
| 13 | TagTaxaCount <= 13.812<br>3.652 < GWidth <= 35.824 | GDist = 0.2622 * TagCount + 0.0032 * TagTaxaCount + 0.01 * GRecom + 8.0E-4 * GBinomP - 0.9294 * lRatio2 - 0.0702 * lRatioM - 0.0639 * GSigSNPNum + 0.0056 * GSigSNPNumBC - 8.0E-4 * GWidth + 4.575 |

| | | |
|---|---|---|
| 14 | TagCount > 8.964<br>LRatio2 <= 2.369<br>GRecom > -2.876 | GDist = -1.4332 * TagCount + 1.2471 * TagTaxaCount + 1.5375 * GRecom - 0.0054 * GBinomP - 0.012 * lRatio2 + 0.4237 * lRatioM - 0.0014 * GSigSNPNum + 0.0099 * GSigSNPNumBC - 1.0E-4 * GWidth - 2.0346 |
| 15 | GWidth <= 41.098<br>TagCount <= 7.2 | GDist = 7.0E-4 * TagTaxaCount + 0.0118 * GRecom + 0.1297 * GBinomP - 0.9814 * lRatio2 - 3.1926 * lRatioM - 0.1694 * GSigSNPNum - 0.0202 * GSigSNPNumBC - 1.0E-4 * GWidth - 10.869 |
| 16 | GWidth > 41.098<br>LRatio2 > 3.698 | GDist = 0.83 * TagCount + 0.0019 * TagTaxaCount + 0.8451 * GRecom - 0.0193 * GBinomP + 0.0033 * lRatio2 - 0.0927 * GSigSNPNum + 0.992 * GSigSNPNumBC + 0.0013 * GWidth + 7.355 |
| 17 | GBinomP > -149.568<br>LRatioM > 2.976 | GDist = -0.1786 * TagTaxaCount + 0.0831 * GRecom + 0.002 * GBinomP - 1.095 * lRatio2 - 0.1365 * lRatioM - 0.105 * GSigSNPNum - 0.0059 * GSigSNPNumBC - 6.0E-4 * GWidth - 14.7727 |
| 18 | GBinomP <= -100.474<br>GSigSNPNum <= 37.834<br>LRatio2 <= 3.011 | GDist = 0.0022 * TagTaxaCount + 4.4913 * GRecom + 3.0E-4 * GBinomP - 0.0366 * lRatio2 + 0.9604 * lRatioM - 0.002 * GSigSNPNum + 0.0137 * GWidth - 24.1363 |
| 19 | GSigSNPNum <= 37.834<br>TagTaxaCount <= 15.066<br>LRatio2 > 1.586<br>GRecom > -3.083 | GDist = 0.0173 * TagCount + 0.0068 * TagTaxaCount + 0.0437 * GBinomP - 0.8483 * lRatio2 + 3.5404 * lRatioM - 0.0011 * GSigSNPNum - 0.4853 * GSigSNPNumBC - 9.7314 |
| 20 | GSigSNPNum > 37.782 | GDist = 3.5696 * GRecom + 0.0015 * GBinomP - 0.028 * lRatioM - 0.0031 * GSigSNPNum - 0.0839 * GSigSNPNumBC - 19.2605 |
| 21 | GSigSNPNumBC > 0.335<br>GRecom > -3.087<br>GBinomP <= -39.619 | GDist = 0.4979 * GRecom + 0.0063 * GBinomP - 1.0617 * lRatioM - 0.0034 * GSigSNPNum - 0.0757 * GSigSNPNumBC - 11.8118 |
| 22 | GWidth <= 2.88<br>GRecom <= -2.988 | GDist = 0.1511 * GRecom + 0.0632 * lRatioM + 0.047 * GSigSNPNum - 0.0044 * GWidth - 10.1197 |
| 23 | | GDist = 7.0127 * GRecom + 27.7579 |

**Supplementary Table 4** Tag mapping accuracy under different thresholds of prediction from M5Rules_G

| Predicting distance threshold | Remaining tags | Mapping accuracy | | | | | | | | | | | |
|---|---|---|---|---|---|---|---|---|---|---|---|---|---|
| | | <10 kb | <20 kb | <50 kb | <100 kb | <200 kb | <500 kb | <1 Mb | <2 Mb | <5 Mb | <10 Mb | <20 Mb | <50 Mb |
| 10 kb | 17.6% | 65.3% | 70.6% | 79.1% | 87.8% | 94.3% | 97.5% | 98.5% | 98.9% | 99.3% | 99.5% | 99.5% | 99.6% |
| 20 kb | 25.1% | 59.4% | 64.7% | 73.5% | 82.9% | 91.1% | 95.9% | 97.6% | 98.4% | 99.1% | 99.3% | 99.4% | 99.4% |
| 50 kb | 35.0% | 52.7% | 58.0% | 67.0% | 76.7% | 85.7% | 92.5% | 95.4% | 97.3% | 98.6% | 99.0% | 99.2% | 99.3% |
| 100 kb | 42.2% | 48.1% | 53.2% | 62.1% | 71.9% | 81.4% | 89.5% | 93.4% | 95.9% | 97.9% | 98.6% | 98.9% | 99.1% |
| 200 kb | 48.4% | 44.6% | 49.4% | 58.1% | 67.7% | 77.5% | 86.4% | 91.2% | 94.4% | 97.1% | 98.2% | 98.6% | 98.9% |
| 500 kb | 54.7% | 41.2% | 45.8% | 54.2% | 63.5% | 73.1% | 82.5% | 88.0% | 92.0% | 95.6% | 97.2% | 98.0% | 98.5% |
| 1 Mb | 58.1% | 39.6% | 44.0% | 52.1% | 61.2% | 70.6% | 80.1% | 85.8% | 90.2% | 94.2% | 96.2% | 97.3% | 98.0% |
| 2 Mb | 60.5% | 38.4% | 42.7% | 50.6% | 59.5% | 68.7% | 78.1% | 83.9% | 88.5% | 93.0% | 95.3% | 96.6% | 97.5% |
| 5 Mb | 63.1% | 37.3% | 41.5% | 49.2% | 57.8% | 66.9% | 76.2% | 82.0% | 86.7% | 91.4% | 94.0% | 95.6% | 96.8% |
| 10 Mb | 64.8% | 36.6% | 40.7% | 48.2% | 56.7% | 65.6% | 74.8% | 80.6% | 85.3% | 90.1% | 92.8% | 94.7% | 96.1% |

UABTs were mapped in all inbred lines. M5Rules_G were trained on 30,000 UABTs. Based on the predicting distance between genetic position and physical position of UABT, a threshold can be set to obtain the desired level of accuracy of mapped tags

**Supplementary Table 5** Tag mapping accuracy under different thresholds of prediction from M5Rules model built in Ames inbreds

| Predicting distance threshold | Remaining tags | Mapping accuracy | | | | | | | | | | | |
|---|---|---|---|---|---|---|---|---|---|---|---|---|---|
| | | <10 kb | <20 kb | <50 kb | <100 kb | <200 kb | <500 kb | <1 Mb | <2 Mb | <5 Mb | <10 Mb | <20 Mb | <50 Mb |
| 10 kb | 9.8% | 63.2% | 68.0% | 76.4% | 86.5% | 93.6% | 98.0% | 98.9% | 99.4% | 99.6% | 99.7% | 99.7% | 99.7% |
| 20 kb | 12.7% | 59.2% | 64.1% | 73.1% | 83.4% | 91.7% | 96.9% | 98.3% | 98.9% | 99.3% | 99.4% | 99.5% | 99.5% |
| 50 kb | 15.9% | 54.0% | 58.8% | 67.4% | 78.1% | 87.7% | 94.4% | 96.9% | 98.3% | 99.1% | 99.3% | 99.4% | 99.4% |
| 100 kb | 18.1% | 50.7% | 55.4% | 63.9% | 74.6% | 84.6% | 92.1% | 95.4% | 97.2% | 98.5% | 98.8% | 99.0% | 99.1% |
| 200 kb | 20.0% | 47.8% | 52.3% | 60.6% | 71.4% | 81.6% | 89.9% | 93.7% | 96.2% | 98.1% | 98.4% | 98.7% | 98.8% |
| 500 kb | 21.8% | 45.3% | 49.7% | 57.6% | 68.1% | 78.4% | 87.4% | 91.8% | 95.0% | 97.1% | 97.7% | 98.2% | 98.4% |
| 1 Mb | 22.9% | 43.9% | 48.1% | 56.0% | 66.3% | 76.5% | 85.8% | 90.5% | 93.9% | 96.2% | 96.9% | 97.5% | 97.8% |
| 2 Mb | 24.1% | 42.5% | 46.7% | 54.4% | 64.5% | 74.6% | 83.9% | 88.9% | 92.4% | 94.8% | 95.7% | 96.5% | 96.9% |
| 5 Mb | 25.7% | 40.7% | 44.8% | 52.2% | 62.1% | 72.1% | 81.4% | 86.5% | 90.1% | 92.8% | 93.8% | 94.6% | 95.1% |
| 10 Mb | 27.0% | 39.2% | 43.2% | 50.4% | 60.0% | 69.8% | 79.1% | 84.2% | 87.9% | 90.8% | 91.9% | 92.8% | 93.4% |

UABTs were mapped in 400 Ames inbred lines. M5Rules model were trained on 30,000 UABTs. Based on the predicting distance between genetic position and physical position of UABT, a threshold can be set to obtain the desired level of accuracy of mapped tags

**Supplementary Table 6** Tag mapping accuracy under different thresholds of prediction from M5Rules model built in NAM inbreds

| Predicting distance threshold | Remaining tags | Mapping accuracy | | | | | | | | | | | |
|---|---|---|---|---|---|---|---|---|---|---|---|---|---|
| | | <10 kb | <20 kb | <50 kb | <100 kb | <200 kb | <500 kb | <1 Mb | <2 Mb | <5 Mb | <10 Mb | <20 Mb | <50 Mb |
| 10 kb | 0.0% | NaN | NaN | NaN | NaN | NaN | NaN | NaN | NaN | NaN | NaN | NaN | NaN |
| 20 kb | 0.0% | NaN | NaN | NaN | NaN | NaN | NaN | NaN | NaN | NaN | NaN | NaN | NaN |
| 50 kb | 0.0% | 0.0% | 0.0% | 0.0% | 0.0% | 100.0% | 100.0% | 100.0% | 100.0% | 100.0% | 100.0% | 100.0% | 100.0% |
| 100 kb | 0.2% | 18.5% | 24.1% | 44.4% | 51.9% | 64.8% | 83.3% | 96.3% | 98.1% | 98.1% | 98.1% | 98.1% | 98.1% |
| 200 kb | 1.5% | 11.7% | 16.1% | 24.8% | 35.3% | 49.3% | 70.4% | 89.7% | 97.5% | 99.3% | 99.5% | 99.5% | 99.5% |
| 500 kb | 4.6% | 8.0% | 10.5% | 15.5% | 23.3% | 34.7% | 56.4% | 77.5% | 91.4% | 98.6% | 99.3% | 99.3% | 99.4% |
| 1 Mb | 9.9% | 6.1% | 8.0% | 11.4% | 17.5% | 26.4% | 43.6% | 63.3% | 81.3% | 93.3% | 96.7% | 98.1% | 99.2% |
| 2 Mb | 15.7% | 4.7% | 6.1% | 9.0% | 13.7% | 21.1% | 36.1% | 53.8% | 71.7% | 88.4% | 94.0% | 96.7% | 98.6% |
| 5 Mb | 23.3% | 3.6% | 4.7% | 6.8% | 10.4% | 16.3% | 28.8% | 43.6% | 59.9% | 78.6% | 87.5% | 92.8% | 97.4% |
| 10 Mb | 27.5% | 3.1% | 4.1% | 6.0% | 9.2% | 14.4% | 25.7% | 39.4% | 54.5% | 73.3% | 83.2% | 89.9% | 95.9% |

UABTs were mapped in 400 NAM inbred lines. M5Rules model were trained on 30,000 UABTs. Based on the predicting distance between genetic position and physical position of UABT, a threshold can be set to obtain the desired level of accuracy of mapped tags

**Supplementary Table 7** Tag mapping accuracy under different thresholds of prediction from M5Rules_GJ

| Predicting distance threshold | Remaining tags | Mapping accuracy | | | | | | | | | | | |
|---|---|---|---|---|---|---|---|---|---|---|---|---|---|
| | | <10 kb | <20 kb | <50 kb | <100 kb | <200 kb | <500 kb | <1 Mb | <2 Mb | <5 Mb | <10 Mb | <20 Mb | <50 Mb |
| 10 kb | 19.4% | 65.0% | 70.7% | 79.2% | 88.2% | 94.8% | 98.0% | 98.8% | 99.3% | 99.4% | 99.5% | 99.5% | 99.6% |
| 20 kb | 27.1% | 59.5% | 64.8% | 73.9% | 84.0% | 92.2% | 97.0% | 98.5% | 99.0% | 99.3% | 99.4% | 99.5% | 99.5% |
| 50 kb | 36.9% | 52.9% | 58.3% | 67.5% | 77.9% | 87.3% | 94.7% | 97.4% | 98.4% | 99.0% | 99.2% | 99.3% | 99.3% |
| 100 kb | 43.4% | 48.8% | 53.9% | 62.9% | 73.3% | 83.1% | 92.0% | 95.9% | 97.6% | 98.6% | 99.0% | 99.1% | 99.2% |
| 200 kb | 49.0% | 45.4% | 50.3% | 59.2% | 69.3% | 79.4% | 88.9% | 93.9% | 96.4% | 98.0% | 98.6% | 98.9% | 99.0% |
| 500 kb | 54.6% | 42.2% | 46.9% | 55.3% | 64.9% | 74.8% | 84.7% | 90.6% | 94.3% | 96.9% | 98.0% | 98.5% | 98.8% |
| 1 Mb | 57.6% | 40.6% | 45.1% | 53.3% | 62.7% | 72.3% | 82.3% | 88.4% | 92.7% | 95.8% | 97.2% | 97.9% | 98.4% |
| 2 Mb | 59.8% | 39.5% | 43.8% | 51.9% | 61.0% | 70.5% | 80.3% | 86.4% | 91.3% | 94.9% | 96.5% | 97.3% | 98.0% |
| 5 Mb | 62.5% | 38.1% | 42.3% | 50.1% | 59.0% | 68.2% | 77.7% | 83.8% | 88.7% | 93.7% | 95.8% | 96.7% | 97.5% |
| 10 Mb | 64.4% | 37.2% | 41.3% | 49.0% | 57.6% | 66.6% | 76.0% | 82.0% | 86.9% | 91.9% | 94.9% | 96.1% | 97.0% |

UABTs were mapped in all inbred lines. M5Rules_GJ were trained on 30,000 UABTs. Based on the predicting distance between genetic position and physical position of UABT, a threshold can be set to obtain the desired level of accuracy of mapped tags

**Supplementary Table 8** Tag mapping accuracy under threshold of models

| Models* | Predicting distance threshold (bp) | Remaining tags | Mapping accuracy | | | | | | |
|---|---|---|---|---|---|---|---|---|---|
| | | | <10 kb | <20 kb | <50 kb | <100 kb | <200 kb | <500 kb | <1,000 kb |
| M5Rules_GJ | 100,000 | 43.4% | 48.8% | 53.9% | 62.9% | 73.3% | 83.1% | 92.0% | 95.9% |
| M5Rules_G | 50,000 | 35.0% | 52.7% | 58.0% | 67.0% | 76.7% | 85.7% | 92.5% | 95.4% |
| M5Rules_J | 100,000 | 21.3% | 30.3% | 35.8% | 45.4% | 58.3% | 74.2% | 89.8% | 95.9% |

*Based on the M5Rules model, three models were trained. M5Rules_GJ was trained for tags mapped by both GWAS and joint linkage mapping. M5Rules_G and M5Rules_J are for GWAS result and joint linkage mapping result, respectively.

**Supplementary Table 9** Enrichment of PAV SNPs in top 0.5% most significant SNPs

| Trait | Expected number of ordinary SNP | Expected number of PAV SNP | Observed number of ordinary SNP | Observed number of PAV SNP | *P*-Value |
|---|---|---|---|---|---|
| Ear height | 335.1 | 589.9 | 306 | 619 | 0.046 |
| Days to anthesis | 335.1 | 589.9 | 310 | 615 | 0.085 |
| Days to silk | 335.1 | 589.9 | 297 | 628 | 0.009 |
| Plant height | 335.1 | 589.9 | 301 | 624 | 0.020 |

Top 0.5% most significant SNPs were selected from GWAS result for each trait. Chi-squared tests were performed to test significance of enrichment. PAV SNPs were significantly enriched in significant GWAS hits in ear height, days to silk and plant height ($P < 0.05$).

**Supplementary References**


1. Elshire, R.J. et al. A Robust, Simple Genotyping-by-Sequencing (GBS) Approach for High Diversity Species. *PLoS ONE* **6**, e19379 (2011).
2. Glaubitz, J.C. et al. TASSEL-GBS: A High Capacity Genotyping by Sequencing Analysis Pipeline. *PLoS ONE* **9**, e90346 (2014).
3. McMullen, M.D. et al. Genetic Properties of the Maize Nested Association Mapping Population. *Science* **325**, 737-740 (2009).
4. Romay, M.C. et al. Comprehensive genotyping of the USA national maize inbred seed bank. *Genome Biology* **14**, R55 (2013).
5. Hung, H.-Y. et al. ZmCCT and the genetic basis of day-length adaptation underlying the postdomestication spread of maize. *Proceedings of the National Academy of Sciences* **109**, E1913–E1921 (2012).
6. Thornsberry, J.M. et al. Dwarf8 polymorphisms associate with variation in flowering time. *Nat Genet* **28**, 286-289 (2001).
7. Christensen, A.H. & Quail, P.H. Structure and expression of a maize phytochrome-encoding gene. *Gene* **85**, 381-390 (1989).
8. Salvi, S. et al. Conserved noncoding genomic sequences associated with a flowering-time quantitative trait locus in maize. *Proceedings of the National Academy of Sciences* **104**, 11376-11381 (2007).
9. Meng, X., Muszynski, M.G. & Danilevskaya, O.N. The FT-like ZCN8 gene functions as a floral activator and is involved in photoperiod sensitivity in maize. *The Plant Cell Online* **23**, 942-960 (2011).
10. Muszynski, M.G. et al. delayed flowering1 Encodes a Basic Leucine Zipper Protein That Mediates Floral Inductive Signals at the Shoot Apex in Maize. *Plant Physiology* **142**, 1523-1536 (2006).
11. Hall, M. et al. The WEKA data mining software: an update. *SIGKDD Explor. Newsl.* **11**, 10-18 (2009).
